# Evaluation of the Effect of an Olive Phenolic Extract on the Secondary Shelf Life of a Fresh Pesto

**DOI:** 10.3390/antiox13010128

**Published:** 2024-01-20

**Authors:** Beatrice Sordini, Stefania Urbani, Sonia Esposto, Roberto Selvaggini, Luigi Daidone, Gianluca Veneziani, Maurizio Servili, Agnese Taticchi

**Affiliations:** Department of Agricultural, Food and Environmental Sciences, University of Perugia, Via San Costanzo s.n.c., 06126 Perugia, Italy; beatrice.sordini@unipg.it (B.S.); stefania.urbani99@gmail.com (S.U.); roberto.selvaggini@unipg.it (R.S.); luigi.daidone@unipg.it (L.D.); gianluca.veneziani@unipg.it (G.V.); maurizio.servili@unipg.it (M.S.); agnese.taticchi@unipg.it (A.T.)

**Keywords:** secondary shelf life, natural product as food additives, olive oil by-product valorisation, bioactive compounds, volatile compounds, lipid oxidation, antioxidant activity, sensory analysis

## Abstract

Recent advances in the olive oil sector aim to develop sustainable strategies for the valorisation of mechanical extraction co-products as a rich source of bioactive compounds with antioxidant and antimicrobial activities. In this work, we studied the effectiveness of a phenolic extract (PE) from olive vegetation water (OVW) as a new antioxidant of natural origin for improving the quality and extending the secondary shelf life (SSL) of a fresh basil pesto sold as a served loose product at the deli counter, simulating the storage conditions after packaging, opening, and serving. For that, the PE was mixed with the oily phase of fresh pesto in two different concentrations and compared to a control pesto (CTRL) made with the addition of common additives (ascorbic acid (E300) and sorbic acid (E200)). The physicochemical parameters, phenolic and volatile composition, sensory profiles, and antioxidant capacity of the experimental pesto samples were evaluated after opening. The results proved that the enrichment with the PE improved the stability of the pesto and, hence, its overall quality. The PE provided higher protection than the CTRL against primary and secondary oxidation at both concentrations tested and delayed the accumulation of the volatile compounds responsible for the ‘rancid’ off-flavour up to 7 days after first opening, while also preserving higher levels of the pesto phytonutrients (such as the rosmarinic, caffeic, and chicoric acids and α-tocopherol). These results show that the generation of food waste in households, catering chains, retail, and/or restaurants can be reduced, improving the sustainability of the food industry and the competitiveness of the olive oil sector.

## 1. Introduction

Fresh basil pesto sauce is a traditional recipe from the Italian region and a valuable source of fibre and health-promoting nutrients, including vitamins and phenolic acids (e.g., rosmarinic acid) [1,2]. Pesto is undoubtedly the best-known ready-made green sauce that does not require any further cooking process, and this ease of use has contributed to its consumption becoming the second-most popular dressing for pasta after tomato [3,4]. The wide variety of products available on supermarket shelves, in chilled cabinets, and in deli counters reflects the large variability of pesto recipes, some of which are traditional or have a protected designation of origin, such as ‘Pesto alla Genovese PDO’ [5] and some non-traditional variants that also differ in terms of their processing and stabilisation methods [6].

In recent years, the consumption of healthy foods typically attributed to the Mediterranean diet has increased, as adherence to this dietary model is widely considered to pre-vent and control various diseases, such as cancer, cardiovascular disease, type II diabetes, and obesity [7]. During the COVID-19 outbreak, this change in people’s lifestyle and dietary habits was reinforced by current evidence and expert opinions recommending the consumption of foods that are rich in antioxidants and vitamins to mitigate the virulence of SARS-CoV-2 [8]. Our view is that fresh basil pesto fits very well with consumer awareness of the importance of health, safety, and convenience in food thanks to its formulation: fresh basil leaves crushed to a creamy consistency and extra virgin olive oil (EVOO), with the addition of garlic, Parmigiano Reggiano and pecorino cheeses, and pine nuts [6]. However, ensuring the quality of pesto and oil-based foods until consumption is a critical factor for the food industry, both in terms of their nutritional value and stability, because they are particularly susceptible to chemical and/or microbiological changes during the manufacturing process and storage before and after opening [9].

Lipid oxidation is the main process responsible for the loss of food quality, leading to the formation and accumulation of free radicals and unpleasant volatile compounds responsible for ‘rancid’ and other off-flavours [10]. An excessive consumption of fat-oxidised products harms human health and increases the risk of cancer, as well as cardio-vascular, metabolic, and neurodegenerative diseases [11]. In addition, microbial alterations can affect the healthy and sensory properties of food and cause toxic infections [12]. Strategies to prevent the development of pathogenic and spoilage microorganisms, minimise lipid oxidation, and inhibit enzymatic and non-enzymatic reactions in foods, especially in pesto, are of significant interest to the industry [4,13]. Among these, different food stabilisation techniques, including thermal and non-thermal treatments [14,15,16], the use of hurdle technology [17], innovative packaging [18], and food additives [4,9,17], have been extensively studied and proposed to delay the above negative phenomena, thus extending the shelf life of pesto. As alternatives to the traditional thermal treatments, reducing the water activity with natural humectants and lowering the pH for a safer and fresher pesto sauce were proposed by Severini et al. [17]. In addition, Fabiano et al. [18] reported that the combination of modified atmosphere packaging (at steady-state, 10% CO_2_, and 90% N_2_) and cold storage (5 °C) had a positive effect on preserving the quality and extending the shelf life of pesto sauce, maintaining its excellent organoleptic appearance and colour for up to 120 days [18]. Nonetheless, the stabilisation of perishable foods with additives is one of the most commonly used methods [13,19,20]. In fact, Turrini et al. [4], evaluating three different alternative formulations of basil-based semi-finished products by changing the preservative (citric acid and ascorbic acid) or introducing blast chilling, pointed out that the use of additives could be excellent for preserving the colour and extending the shelf life of these products until three months of storage at refrigeration temperature (4 °C). Although food additives perform many important technological functions, including protection against oxidation, the inhibition or inactivation of spoilage and enzymes, and the improvement of sensory properties (colour, aroma, taste, and texture) [21], their use has been established by legal regulations [22]. Synthetic additives are often perceived negatively by consumers due to their toxicity and carcinogenic effects on various organs and body systems [23,24], and their replacement is therefore highly sought-after.

The growing awareness of environmental issues and dietary influence on human health is arousing consumer interest in minimally processed, healthy foods or those containing natural food additives and/or ingredients, referred to as ‘clean label’ foods [25]. It is well known that there are many additives of natural origin that are permitted in limited doses, often linked to the admitted daily intake (ADI), and whose efficacy is not always satisfactory under the conditions of use. Trends for the food industry can balance the needs of technological purpose (e.g., efficacy) with those of the market (e.g., confidence). Sorbic acid, for example, is a natural preservative (E200) that is widely used and can be consumed throughout the day in foods containing it up to doses close to the limit (11 mg per day per kg of body weight) [26]. On the other hand, ascorbic acid (E300) has a distinctive ‘sour’ taste, which can therefore affect the flavour of the product in an unpleasant way [24,27,28]. Thus, the challenge is to find natural food additives from plant sources or food industry by-products that can replace or partially substitute synthetic ones [25]. Among these, the newest antioxidants obtained from the food industry by-products have attracted much interest and appear to be the most promising, as they embrace the policies for transitioning towards a circular economy and sustainability [27,29]. EVOO and its by-products of the mechanical extraction process (olive pomace and olive vegetation water (OVW)) are a rich and exclusive source of phenolic compounds (especially the derivatives of secoiridoids) with recognised biological and sensory properties [30]. EVOO contains only 2% of the entire phenolic content of olive fruit, while significant amounts are also found in OVW and pomace, making them highly polluting forms of waste in the Mediterranean area [31]. An eco-friendly membrane filtration system was applied at an industrial scale plant to completely eliminate the pollution load of OVM and to recover target bioactive compounds without the use of chemical solvents. The phenolic extract (PE) obtained using green technology is currently considered a promising new natural antioxidant with biological activities and technological functionality, helpful in satisfying consumer demand and reducing environmental issues [31]. The chemical composition of the PE and its efficacy when applied as a natural preservative or bioactive ingredient to improve quality and extend shelf life have recently been studied by several authors on EVOO [31,32] and through different food matrices [33,34,35,36,37,38].

Despite the large presence of fresh basil pesto on the market, there is a lack of studies on its overall quality and its evolution during the primary and secondary shelf lives (SSLs) after opening the package. To the best of our knowledge, only one previous study examined eight commercial pesto sauces for their pH, colour parameters, volatile compounds, and microbiological and sensory profiles, simulating household conditions [9]. In addition, consumer concerns about their safety and the increasing demand for ‘clean label’ products have led the food industry to search for novel antioxidants to partially or fully replace synthetic ones. There is limited research on the use of natural antioxidants from plant and/or agro-industrial by-products to extend the shelf life of pesto sauce [37]. In this work, we characterised fresh pesto sauce enriched with PEs from OVW in terms of physicochemical parameters, phenolic and volatile composition, sensory profiles, and antioxidant activity, and its evolution was monitored during the SSL, simulating the real-time storage conditions of a served loose product at the deli counter (i.e., every day, a quantity of pesto sauce is scooped from the opened pack, increasing the volume of headspace occupied by air), as this is a common sales method in Italian food stores. This aspect is particularly sensitive, because in these severe conditions, the quali-quantitative modifications that affect the product quality occur more quickly than in a sealed and correctly stored package; this experimental approach also makes it possible to estimate the food shelf life, once opened, under home storage conditions. The main objective was to evaluate the antioxidant effect of the PE from OVW at two different concentrations as a ‘clean label’ ingredient to slow down the qualitative decline of fresh pesto up to 7 days after opening, in comparison with the most common preservatives used in the food industry (ascorbic acid (E300) and sorbic acid (E200) in combination).

## 2. Materials and Methods

### 2.1. Chemicals

*p*-HPEA, 3,4-DHPEA, verbascoside, rosmarinic acid, and α-tocopherol were purchased from Merk (Milan, Italy). The secoiridoid derivatives (i.e., oleacein (3,4-DHPEA-EDA) and oleocanthal (*p*-HPEA-EDA)) were obtained as reported in the previous work of Taticchi et al. [39]. Then, 2,4,6-tripyridyls-triazine (TPTZ), 6-hydroxy-2,5,7,8-tetramethyl-chroman-2-carboxylic acid (Trolox), 2,2-diphenyl-1-picrilidrazil (DPPH˙), and butylated hydroxytoluene (BHT) were purchased from Merk (Milan, Italy). Formic acid, n-hexane, phosphoric acid, hydrochloric acid, methanol, ethanol, 2-propanol, and acetonitrile were supplied by VWR (Milan, Italy). HPLC-grade methanol and water for HPLC-MS were supplied by Carlo Erba (Milan, Italy). Finally, 4-methyl-2-pentanol, as an internal standard for pesto volatile compounds, was purchased from Sigma-Aldrich (Milan, Italy).

### 2.2. OVW Phenol Extract

A PE was obtained through three-stage membrane filtration from fresh OVW, as previously reported by Esposto et al. [32]. Hydrophilic phenols from the PE were determined through high-performance liquid chromatography (HPLC) analysis using an Agilent Technologies System mod. 1100 (Santa Clara, CA, USA), as previously described by Selvaggini et al. [40]. A C18 column of Spherisorb ODS-1, 250 × 4.6 mm, with a particle size of 5 μm (Phase Separation Ltd., Deeside, UK), was used. The phenolic compounds were detected using a diode array detector (DAD) at a wavelength of 278 nm. The chemical composition of the PE, which was used to prepare the experimental pesto samples, is listed in Supplementary Table S1. The purity of the PE was 74%, and the total concentration of phenolic compounds was 748.3 ± 3.3 mg/g, of which 636.0 mg/g was oleacein (3,4-DHPEA-EDA), 80.9 mg/g hydroxytyrosol (3,4-DHPEA), 14.6 mg/g tyrosol (*p*-HPEA), 10.4 mg/g verbascoside, and 5.7 mg/g oleocan-thal (*p*-HPEA-EDA).

### 2.3. Preparation of Phenol-Enriched Pesto Samples

The fresh pesto samples were kindly manufactured by a local company (Perugia, Italy). The basic pesto recipe was as follows: thermally stabilised basil semi-finished product, Grana Padano cheese, Pecorino cheese, pine nuts, nuts, salt, garlic, and sunflower oil. Based on this recipe, three experimental samples were prepared: (i) control (CTRL) plus 0.06 g of ascorbic acid/kg of pesto and 1 g of sorbic acid/kg of pesto; (ii) phenolic extract pesto number one (PEP1) plus PE1, corresponding to 250 mg of phenols/kg of pesto; and (iii) phenolic extract pesto number two (PEP2) plus PE2, corresponding to 500 mg of phenols/kg of pesto. The two concentrations of addition (PE1 and PE2) were chosen in a range of total phenol content comparable to those occurring in good-quality EVOOs. To achieve the final phenolic compound concentrations of 250 mg and 500 mg of phenols/kg of pesto, 801.1 mg and 1602.1 mg of PE were weighed and dissolved in the oil phase. The manufacturing process of the fresh pesto on an industrial scale consisted of mixing the ingredients in a grinder, cold filling at 10 ± 2 °C, and packaging in a polypropylene plastic bucket (1 kg). The end product had an estimated shelf life of 5–6 days after opening or 120 days if unopened at the storage temperature of 4–5 °C, according to the manufacturer’s indications.

### 2.4. Experimental Set-Up for Real-Time Storage of Deli Counter Food Products

To simulate the real-time storage conditions of a served loose product at the deli counter, three plastic buckets were opened and stored for 7 days in a climate cell at 4 ± 2 °C under fluorescent light (600 lx) and air exposure per 12 h a day. After opening, each pesto sample (CTRL, PEP1, and PEP2) was sampled at a fixed storage time (immediately after opening (day 0), and 1, 2, 3, 6, and 7 days of storage after opening). On those days, three aliquots (25 g) of each pesto sample were removed (at 9:00 a.m., 12:00 p.m., and 3:00 p.m.) to increase the headspace in the bucket during the experiment, and at 6:00 p.m., a sample of 50–75 g was taken and subjected to physicochemical and sensory analysis.

### 2.5. Analytical Determination

Analytical determinations were carried out for each pesto sample (CTRL, PEP1, and PEP2) at all six time points: 0, 1, 2, 3, 6, and 7 days of storage after the plastic bucket opening, unless otherwise stated.

#### 2.5.1. Extraction and Evaluation of Phenolic Compounds

The extraction of individual phenolic compounds was performed as previously de-scribed by Miraglia et al. [33], with some modifications. Briefly, pesto (1 g) was homogenised with 10 mL of a methanol: water solution (80:20, *v*/*v*) (containing 0.2% (*w*/*v*) butylated hydroxytoluene (BHT)) using an Ultraturrax T25 IKA (Janke and Kunkel, Staufen, Germany) at 17,000 rpm for 3 min. The homogenate was centrifuged at 9000 rpm for 10 min, and the supernatant was transferred into a 100 mL flask and collected. The extraction procedure was carried out twice. The supernatant was collected and dried at 37 °C using a rotavapor. The dried product (residue) was dissolved in 1 mL of methanol and filtered using 0.2-μm syringe filters made of cellulose acetate (CA) (Carlo Erba, Italia). The purified extract was then subjected to HPLC-DAD analysis using the same equipment and conditions as for the PE analysis [33] and UHPLC-DAD-Q-TOF/MS. Each analysis was carried out in duplicate. The identification of the phenolic compounds found in the pesto, mainly derived from basil, was performed through UHPLC-DAD-Q-TOF/MS analysis (Table S2 [41]) using the equipment and the column described in a previous study [42] with an ultra-high-performance liquid chromatography system (UHPLC, Agilent Technologies, mod. 1260 Infinity, Palo Alto, CA, USA) coupled to a quadrupole time-of-flight (Q-TOF) mass spectrometer with electrospray ionisation source (dual ESI, model Agilent 6530 Accurate-Mass Q-TOF LC/MS, Agilent Technologies, Palo Alto, CA, USA). The column used was a Zorbax Eclipse Plus C18 100 mm × 2.1 mm, 1.8 μm (Agilent Technologies, Palo Alto, CA, USA). The injected sample volume was 2 μL, and elution was performed at a flow rate of 0.270 mL/min using water with 0.1% formic acid as eluent A and acetonitrile with 0.1% formic acid as eluent B. The elution gradient was as follows: 86% A/14% B; at 8 min, 60% A/40% B; at 16 min, 50% A/50% B; at 11 min, 65% A/35% B; at 16 min, 50% A/50% B; and at 20 min, 0% A/100% B, and was maintained for 10 min. Then, the system was restored to its initial conditions of the analysis after 3 min and allowed to equilibrate with a follow-up time of 5 min. The total time of this analysis was 38 min, while the acquisition time was 30 min. The mass spectrum was acquired through ESI ionisation in negative mode in the m/z range of 50–1700 with a scan rate of 1.5 spectrum/s, simultaneously infusing in addition to the eluent from the HPLC system (via the first nebuliser), with the two reference masses (via the second nebuliser) having m/z values of 112.985587 and 980.016375. The parameters of the dual ESI source were set to the following conditions: gas temperature, 335 °C; flow of drying gas, 10 L/min; nebuliser gas pressure, 35 psig; capillary voltage, VCap 3500 V; fragmenter, 120 V; skimmer, 65 V; and Octapol 1 RF, 750 V. Under the above chromatographic conditions, the data were recorded in all-ion mode MS/MS by recording the chromatogram in full scan and MS/MS with a collision energy of 15 V and 25 V, respectively. Agilent MassHunter B. 06.00 software was used to control the instruments and data. Several online libraries of MS and MS/MS spectra (METLIN, Human Metabolome Database, or HMDB and mzCloud) and the available literature were used for compound identification [43]. The quantitative assessment of phenols present in the pesto (extracted from basil) was performed using the rosmarinic acid calibration curve, and the results were expressed in mg/kg.

#### 2.5.2. Determination of Free Acidity, Peroxide Value, Fatty Acid Composition, and α-Tocopherol Content of the Oil Extracted from Pesto

For oil extraction, 10 g of pesto were mixed with 30 mL of hexane, homogenised with the Ultraturrax T 25 IKA (Janke and Kunkel, Staufen, Germany) for 1 min at 17,000 rpm, and then filtered with a paper filter. This process was repeated twice. The filtered homogenate was collected and dried at 35 °C with a rotavapor until complete evaporation of the solvent. The free acidity (FA) (%) and peroxide value (PV) (milliequiv. of O_2_/kg of oil) were determined for the oil extracted from the pesto according to the AOCS (American Oil Chemist’s Society) official methods Cd 3d-63 and Cd 8-53, respectively [44,45]. The fatty acid composition of the oil extracts from pesto was analysed through gas chromatography (GC) using a Dani Master GC-FID (DANI Instruments, Milan, Italy) according to the method described in EU Reg. 2022/2104 [46]. For the evaluation of α-tocopherol, an aliquot of 1 g of the oil extract was dissolved in 10 mL of n-hexane, filtered with 0.2-μm PVDF syringe filters (Carlo Erba, Milano, Italia), and then its concentration was determined through HPLC with a diode array and fluorescence detectors. The HPLC analysis was performed with the same equipment (HPLC-DAD-FLD) as used for the analysis of PE phenolic compounds (Section 2.5.1.), the same procedure published by Psomiadou and Tsimidou [47], and successively modified by Esposto et al. [32]. Briefly, the column used was a normal-phase Lichrospher Si 60, 250 × 4 mm, with a particle diameter of 5 μm (Merk KgaA, Darmastadt, Germany). The injection volume was 50 μL, and the eluent flow was 1.3 L/min using a mixture of n-hexane/2-propanol (99.5:0.5 *v*/*v*) (A) and n-hexane/2-propanol (70:30 *v*/*v*) (B). The gradient was as follows: 100% A/0% B for 2 min; in 8 min, 95% A/5% B; in 5 min, 25% A/75% B for 4 min; and back to baseline in 3 min with a follow-up time of 5 min. The total run time was 35 min. α-tocopherol was detected at an excitation wavelength of 295 nm and at an emission wavelength of 300 nm. The quantitative evaluation of α-tocopherol in the oil extracted from pesto was performed by plotting the calibration curve of the pure compound as a standard, and the results were expressed in mg/kg.

#### 2.5.3. Evaluation of Antioxidant Potential

The antioxidant power of the pesto samples was evaluated by antiradical activity through the 2,2-diphenyl-1-picrilidrazil (DPPH˙) assay, according to the method described by Brand-Williams et al. [48]. Briefly, 200 μL of the methanolic extract of the pesto, the same extract used for the HPLC analysis of the phenols, was added to 3.8 mL of the methanolic DPPH˙ solution (25 mg/L). The absorbance of the reaction mixture was determined after 20 min at room temperature and in the dark at 515 nm. The analyses were performed using a Cary 100 Scan UV–visible spectrophotometer (Varian, Walnut Creek, CA, USA), and the results were expressed in μmol Trolox equivalent (TE) in mL of the sample using the Trolox calibration line (0.01–0.1 μmol).

#### 2.5.4. Evaluation of Volatile Compounds

The volatile compounds were detected through mass spectrometry (MS) analysis coupled to gas chromatography (GC) through the headspace using solid-phase microextraction (SPME). For sampling the volatile compounds in the headspace, SPME was applied as follows: 2 g of pesto were mixed with 2 mL of a saturated aqueous NaCl solution and placed in a 20 mL vial. Then, 20 μL of 4-methyl-2-pentanol were added as an internal standard at a concentration of 750 µg/L, and the vial was subsequently tightly sealed with a polytetrafluoroethylene (PTFE) septum and placed in the autosampler. SPME sampling of volatile compounds and GC/MS analysis were performed using the same equipment and analytical conditions described by Esposto et al. [42]. The volatile compounds were identified by comparing the mass spectra and retention times we obtained with those of pure analytical standards and with the spectra of the NIST 2014 library. The volatile compounds were quantified and expressed in µg/kg by comparing the area of the extracted ion of each peak with the area of the peak of the internal standard ion (4-methyl-2-pentanol), as reported by Xiao et al. [49].

#### 2.5.5. Measurement of Colour Parameters and pH

The colour parameters were measured using a Cary 100 Scan UV–visible spectrophotometer (Varian, Walnut Creek, CA, USA). The values of the coordinates CIE-Lab and CIE-LCh, L* (lightness), a* (redness/greenness), b* (yellowness/blueness), C* (chroma), and h* (hue), which are related to the psychophysical properties of colour [50], were recorded. These coordinates were calculated based on the data obtained with the standard illuminant D65 (which simulates daylight; colour temperature: 6504 K) in the scanning range of 380–770 nm, a data recording interval of 5 nm, and an observer with an angle of 10°. The analyses were performed in duplicate using 2-mm glass cuvettes. All recorded data were processed using Cary WinUV Colour software (Version 4.20 (468)). The colour parameters of the samples CTRL, PEP1, and PEP2 were used to calculate the Euclidean distance (ΔE) between two points in three-dimensional space, while the differences in the browning index (BI) were calculated using the equations of Nicosia et al. [9]. The pH values were recorded using a pH meter (Mettler Toledo©, Barcelona, Spain). All measurements were carried out in duplicate.

### 2.6. Sensory Analysis

The pesto samples (0 and 7 days of storage after opening) were subjected to a descriptive–quantitative sensory analysis by a panel of experienced judges (six females and four males with ages ranging from 25 to 60) previously trained for sensory analysis, according to the ISO 8586: 2012 standard [51]. The temperature of the tasting room was 20 °C. The samples were placed on white plates coded with random three-digit numbers. The panellists received and tested the samples in a balanced order, reporting their quantitative evaluations on an unstructured scale, with a 9-cm long line for each attribute. The coefficient of variation evaluated for each attribute was less than or equal to 20%, considering the mean value of the intensity provided by the 10 panellists for each attribute. The attributes were divided into different categories, namely sight (‘green’, ‘yellow’, and ‘brown’), smell (‘fresh/fragrant basil’, ‘cheese’, and ‘pine nuts/walnuts’), and taste (‘sour’, ‘bitter’, ‘salty’, and ‘metallic’). In addition, trigeminal sensation (‘pungent’), the final sensation (‘overall pleasantness’), and the off-flavour of ‘rancid’ were also included. These results were added to the multivariate statistical analyses (principal component analysis (PCA) and partial least squares projections to latent structures (PLS) models).

### 2.7. Statistical Analysis

The physicochemical and sensory data were analysed using one-way analysis of variance (ANOVA) using Tukey’s test (*p* < 0.05). The software used was SigmaPlot v. 12.3 (Systat Software Inc., San Jose, CA, USA). The chemometric package SIMCA 13.0 (Umetrics AB, Umeå, Sweden) was applied to perform PCA and PLS analyses of the instrumental and sensory data.

## 3. Results and Discussion

### 3.1. Evolution of Phenolic Compound Content

Table 1 shows the phenolic concentrations of the three experimental pesto samples analysed during the 7 days of storage. At 0 days, corresponding to the package opening time, the content of hydrophilic phenols added with the PE was 236.6 ± 2.6 mg/kg and 435.5 ± 4.6 mg/kg in PE1 and PE2, respectively, corresponding to the samples of PEP1 and PEP2, respectively, confirming the high solubility of the PE (Table S3). As expected, no traces of hydrophilic phenols were found in the CTRL (Table S3). In particular, the recovery percentage of hydrophilic phenols obtained for PEP1 and PEP2 was very high (95% and 87%, respectively), with only a minimal amount of their concentration lost during the mixing phase of the ingredients, probably partially due to their oxidation. Among these, in terms of individual phenolic species, the oleuropein derivatives (hydroxytyrosol (3,4-DHPEA) and oleacein (3,4-DHPEA-EDA)) were the most abundant in the PEP1 and PEP2 samples. Their percentage of the total phenolic fraction was 18% (43.6 ± 2.5 mg/kg) and 75% (185.8 ± 0.6 mg/kg) in the PEP1 sample and 17% (76.0 ± 1.1 mg/kg) and 79% (343 ± 4.4 mg/kg) in the PEP2 sample, respectively. The two concentrations of PE, which were sensory acceptable according to previous studies [33,35], were capable of limiting or delaying the primary and secondary oxidation processes and microbial spoilage in various foods (e.g., milk [34], meat [35,36,52], and seafood [33]), ensuring their oxidative and microbiological stability during storage.

In addition to cheese (Parmigiano Reggiano and Pecorino Romano), pine nuts, nuts, and garlic, basil leaves are the main ingredients of the traditional recipe for green pesto sauce [2]. It is important to note that basil is recognised as a valuable source of bioactive compounds, mainly phenolic acids (especially rosmarinic acid, caffeic acid, caftaric acid, and chicoric acid), which contribute to its strong antioxidant capacity and health-promoting and sensory properties [53]. At package opening (day 0), rosmarinic acid was the most abundant phenolic acid, accounting for 46% (169.6 ± 2.4 mg/kg), 44% (162.6 ± 1.8 mg/kg), and 46% (172.5 ± 1.7 mg/kg) of the total content in the samples CTRL, PEP1, and PEP2, respectively. The caffeic acid content accounted for 21–25% of the total phenols from basil and ranged from 79 mg/kg to 92 mg/kg, followed by chicoric acid, salvianic acid, and caftaric acid. These results agree with those of some authors who reported the phenolic profile of basil leaves [2,43]; however, to our knowledge, this study is the first to characterise the phenolic profile of pesto sauce with PE enrichment and without, which was industrially manufactured. Since the phenols in basil are particularly susceptible to degradation [54], it is not surprising that the concentrations of phenolic acids we found (ranging from 363 mg/kg to 373 mg/kg) were lower than those measured in basil leaves (between 88 and 1185 mg/kg) by Ciriello et al. [2], and were higher than those detected by De Bruno [37] on fresh and thermally stabilised commercial pesto (230 mg/kg and 250 mg/kg, respectively). This wide variability in phenolic compounds could be partially attributed to the different genotypes or growing conditions, as well as the influence of the processing method; thus, the low content in commercial basil products could be due to thermal treatments, storage conditions, and/or the drying process [6].

During the SSL, part of the phenol content is depleted, as shown in Table 1 and Figure S1. The total phenolic content (expressed as the sum of phenols from basil and the sum of phenols from the PE) significantly decreased (*p* < 0.05) in all the pesto samples, with a loss of 29% (from 364.4 ± 3.3 to 259.8 ± 1.4 mg/kg) in the CTRL, 25% (from 599.5 ± 3.6 to 449.0 ± 3.3 mg/kg) in PEP1, and 18% (from 808.8 ± 5.3 to 662.5 ± 5.2 mg/kg) in PEP2, respectively. At the beginning of the storage period, in the CTRL sample, the concentration of phenols from basil showed a negligible decrease; however, after the third, sixth, and seventh days after opening, it decreased faster by 9.5%, 22.0%, and 28.7%, respectively. These results can be explained by the fact that ascorbic acid is rapidly oxidised by the molecular oxygen present in the headspace of the packaging and contact with the pesto, decreasing the protective effect against the oxidation of the various phytonutrients it contains. This finding aligns with the study by Shen et al. [55] that pointed out that the oxidation reaction is responsible for most of the loss of ascorbic acid activity in foods.

Observing the behaviour of the individual phenolic fractions, the CTRL samples had a lower content of phenols from basil than the PE-enriched ones at all times tested. When stored for 7 days after opening, the reduction in phenols from basil in CTRL, PEP1, and PEP2 was 28.7% (259.8 ± 1.4 mg/kg), 16.3% (303.5 ± 3.0 mg/kg), and 9.4% (338.2 ± 3.6 mg/kg), respectively. In the PEP1 and PEP2 samples, higher amounts of phenols from basil remained than in the CTRL sample (16.8% and 30.2%, respectively), confirming that the PE was also effective in preventing their oxidation. This effect was particularly evident for the contents of rosmarinic and chicoric acids. Their concentration was lower in the CTRL sample than in the phenol-enriched ones (15.3% and 48.3% lower than those found in PEP1, and 40.5% and 61.1% lower than those found in PEP2, respectively). Regarding the evolution of hydrophilic phenols from the PE during the SSL (Table 1 and Figure S1), the percentage retained was 38% and 25% of the amount added to PEP1 and PEP2, respectively. The oleuropein derivatives (hydroxytyrosol (3,4-DHPEA) and oleacein (3,4-DHPEA-EDA)), which represented 96% of the initial phenols in both samples enriched with the PE, on average, showed a similar decrease in concentration as the total phenols of this fraction, confirming their faster involvement in limiting the oxidation rate of the pesto samples. The 3,4-DHPEA-EDA concentration decreased by less than 10% (168.7 ± 2.0 mg/kg) and 6% (24.3 ± 0.7 mg/kg) in PEP1 and PEP2, respectively (after the first 3 days). After the sixth and seventh days of storage after opening, this compound more abruptly disappeared in PEP1 (between 27% (135.8 ± 3.9 mg/kg) and 45% (102.7 ± 1.3 mg/kg) of the initial concentration of the secoiridoid derivatives) than in PEP2, where its decrease was limited to 11% (306.8 ± 1.0 mg/kg) and 25% (257.9 ± 3.7 mg/kg), respectively. Conversely, at 7 days of storage after opening, the concentration of 3,4-DHPEA was partially lost in PEP1, while it decreased more in PEP2 (17% (35.5 ± 0.6 mg/kg) and 33% (50.5 ± 0.8 mg/kg) of the initial concentration of hydrophilic phenols, respectively); however, a significant increase (*p* < 0.05) was observed in both pesto samples enriched with the PE (after 3 days and 6 days of storage after opening for PEP1 and PEP2, respectively). This behaviour of 3,4-DHPEA is probably due to its lower involvement in oxidation reactions compared to 3,4-DHPEA-EDA, so it only comes into play when the latter is constantly decreasing, and, on the other hand, a possible simultaneous increase due to the effect of hydrolysis of the same 3,4-DHPEA-EDA. As reviewed by Servili et al. [30], the degradation mechanism of 3,4-DHPEA-EDA includes enzymatic and non-enzymatic oxidation and hydrolysis reactions. The smoother degradation of 3,4-DHPEA in the first days of storage and the increase in concentration at 3 days and 6 days of storage after opening depends on the simultaneous occurrence of the hydrolysis of 3,4-DHPEA-EDA, which releases 3,4-DHPEA in free form and limits its loss [56]. In contrast, the *p*-HPEA and verbascoside concentrations did not decrease significantly (*p* < 0.05) as a function of storage time, as already found in other studies on the shelf life of food matrices [33,35]. This effect was more pronounced in the pesto with the highest PE enrichment level (PEP2). This finding supports the assumption of some prior research that ligstroside derivatives contribute to the stability of oils [57] and other food matrices [33,35], but to a lesser extent than oleuropein derivatives [33,35,57,58]. Our results clearly show the superior effectiveness of the PE in preserving the native phenolic compounds of the pesto compared to ascorbic acid (added in the CRTL sample) and that its efficacy is dose dependent. This observed protective behaviour of PE phenols towards pesto phytonutrients during storage is very similar to that described by Taticchi et al. [39] for the same PE species towards phenols and carotenoids in a tomato sauce during cooking. This aspect is particularly interesting, as caffeic acid derivatives (e.g., rosmarinic acid, caftaric acid, caffeic acid, and chicoric acid) exhibit strong antioxidant, anti-inflammatory, antiviral, and immunostimulant properties [59,60,61]. Moreover, these compounds can be considered powerful multitargeting agents against human immunodeficiency virus type 1 (HIV-1) [53,59] and some other diseases, including diabetes and skin melanoma [43]. In addition, the total residual concentration of bioactive phenols added to pesto sauces with the PE, after 7 days of storage after opening, can still allow for the uptake of more than 14 mg/100 g of product (PEP1) and more than 30 mg/100 g (PEP2) of bioactive molecules claimed to be responsible for the protection of blood lipids against oxidative stress and, thus, the risk of cardiovascular disease, at a minimum intake of 5 mg hydroxytyrosol and its derivatives per day according to Commission Regulation (EU) 432/2012 [62].

### 3.2. Evolution of Free Acidity, Peroxide Value, Fatty Acid Composition, and α-Tocopherol Content of Oil Extracted from Pesto

To better compare the performance of the additives used in this study to slow down the natural oxidation processes of pesto sauce, the oil extracted from the CTRL, PEP1, and PEP2 samples was analysed in terms of its chemical quality indices (fatty acid composition (Table S4), free acidity (FA), peroxide values (PVs) (Table 2), and α-tocopherol content (Table 3).

The lipid content in pesto is about 60 g/100 g of product. The most abundant fatty acids were linoleic acid (about 47%) and oleic acid (about 33%), followed by palmitic acid (14%), stearic acid (4%), and traces of linolenic acid, arachidic acid, palmitoleic acid, margaric acid, and cis-11-eicosenoic acid (Table S4). The addition of PE1 and PE2 to the lipid phase of the pesto did not change the fatty acid composition, maintaining the typical composition of sunflower oil in the pesto sauce (Table S4). The percentages of saturated fatty acids (SFAs), monounsaturated fatty acids (MUFAs), and polyunsaturated fatty acids (PUFAs) were 19%, 33%, and 48%, respectively, and no differences were observed between the pesto samples analysed during the real-time shelf life test (Table S4). Comparing our results with those of Zardetto and Barbanti [6], similar and often higher values were found. Moreover, the values of FAs (%) did not change over time and ranged from 0.55 ± 0.01 to 0.63 ± 0.02, as indicated in Table 2. Conversely, in this type of product, the lipid oxidation reaction occurs faster, mainly due to the high surface area-to-volume ratio, which favours the interactions between the lipid phase and the pro-oxidant factors (oxygen availability, temperatures, and exposure to light) [63]. In general, the composition of fatty acids (especially PUFAs) makes this elective oxidation substrate readily accessible to autoxidation [64]. Singlet oxygen is a short-lived ROS that directly oxidises unsaturated fatty acids whose primary reaction products are hydroperoxides [64]. Therefore, the oil phase of the pesto samples, in which PUFAs were the predominant class of fatty acid composition and the oxygen concentration in the headspace increased with each sampling, was susceptible to oxidation processes under the simulation of the sale and storage conditions of a served loose product at the deli counter. This process was confirmed with the PV results (Table 2). Indeed, the PV for all three samples increased significantly (*p* < 0.05) as a function of the storage days after opening and the different formulations. In the CTRL and PEP1 samples, the PV increased starting from the third day of storage after opening, reaching an increase of 91% and 90% on the seventh day, respectively. Significantly lower PV levels (*p* < 0.05) were found in PEP2 oil compared to those of CTRL and PEP1, ranging between 8.9 and 12.7 meq O_2_/Kg of oil, with a maximum increase of 42%. Similar results were obtained by Zardetto and Barbanti [6], who developed a shelf life model for fresh green pesto during 45 days of storage at three different temperatures (4 °C, 8 °C, and 12 °C) using an accelerated test. These authors observed that the PV increased with storage time, and this parameter could be a representative marker of the deterioration of fresh green pesto during its shelf life [6].

The health-promoting effects of α-tocopherol, including antioxidants and vitamin E, are well known [65]. Thanks to its hydrophobic nature, α-tocopherol plays an important role in stabilising the lipid fraction and acts as a chain-breaking antioxidant [66]. Table 3 shows the α-tocopherol content of the oil extracted from the pesto samples at opening (day 0) and its evolution during the 7-day storage period after opening. In the just-opened pesto, no significant differences (*p* > 0.05) were found in the α-tocopherol content between the CTRL samples and those enriched with the PE. After 7 days of storage after opening, a significant (*p* < 0.05) decrease of 4.2% was observed in the CTRL sample, while the decrease was limited to 3.5% and 2.7% for PEP1 and PEP2, respectively. Although at the end of the 7 days of the SSL, there were no statistically significant differences between the CTRL, PEP1, and PEP2 samples, a greater efficacy of the PE (samples PEP1 and PEP2) can be seen in slowing down the degradation of α-tocopherol compared to that observed for the ascorbic acid (CTRL sample). The pesto sample with a higher content of antioxidants (especially the oleuropein derivatives, such as 3,4-DHPEA-EDA) delayed the oxidative and photo-oxidative phenomena, which are strongly negative on food quality. These results are consistent with the recent studies by Esposto et al. [57,58], which demonstrated the faster involvement of oleuropein derivatives in limiting the decay phenomena of the EVOO, not only slowing down the production of free radicals and volatile compounds responsible for the ‘rancid’ off-flavour [67] but also being able to protect other, less active antioxidants and healthy components such as vitamin E. All of these activities are more evident the higher the initial concentration of 3,4-DHPEA-EDA was [57]. These results are consistent with those reported in the literature demonstrating a synergistic interaction between α-tocopherol (hydrophobic vitamin E) and various food additives to delay the oxidation of base–lipid foods [68]. In the food industry, the best-known and most used combination to prevent oxidation in foods is α-tocopherol and ascorbic acid [69]. Baldioli et al. [70] investigated the synergistic effect of derivatives of secoiridoids and α-tocopherol to counteract oxidative processes in EVOO, while Panya et al. [68] reported that rosmarinic acid showed a strong synergistic interaction with α-tocopherol in O/W emulsions. Since PE phenols reduced the degradation of rosmarinic acid, they exerted a better protective function in maintaining α-tocopherol content than ascorbic acid. Interestingly, this effect is very important for consumer well-being due to the antioxidant and vitamin-like properties of α-tocopherol [65,66].

### 3.3. Evolution of the Antioxidant Potential of the Pesto

In this study, the antioxidant activity of the experimental pesto samples at package opening (day 0) and after 1, 2, 3, 6, and 7 days of storage was measured using the DPPH˙ assay. As shown in Table 4, while at day 0, there were no significant differences among the three experimental samples, during the SSL, the antioxidant activity was significantly (*p* < 0.05) influenced by product formulation. Under these severe storage conditions (when light and oxygen exposure were not limited), the antioxidant capacity of the CTRL samples was 1.9 and 1.8 times lower than in the phenol-enriched ones (PEP1 and PEP2, respectively). In particular, for the CTRL, PEP1, and PEP2 samples, the initial value of antioxidant activity was 4.59 ± 0.08, 4.67 ± 0.04, and 4.78 ± 0.1 (μmol TE/g f.w.), and after 7 days, it was reduced to 2.14 ± 0.11, 3.81 ± 0.01, and 4.17 ± 0.01 (μmol TE/g f.w.), respectively. The antioxidant activity of all the samples decreased significantly (*p* < 0.05) during the SSL; it was halved in the CTRL and lower in the PEP1 and PEP2 samples (18% and 12%, respectively). Our results provided information about the contribution of phenols and their antioxidant potential. The DPPH˙ values seem to perfectly match the different phenolic concentrations detected in the pesto samples on the seventh day after opening. The CTRL had the lowest phenolic content and also showed the lowest antioxidant activity. In our study, the antioxidant activity values measured using the DPPH˙ assay were generally lower than those found in the literature and were often not interchangeable, as they depend on different factors related to the basil leaves and the method used. The results of Nguyen et al. [71] showed that the antioxidant capacity values of the basil leaves determined using the same assay ranged from 2.4 mmol TE/100 g d.w. for Dark Opal basil to 6.7 mmol TE/100 g d.w. for Genovese basil, one of the most commonly used basil varieties for the preparation of pesto [2].

A plot of DPPH˙ values as a function of the total phenolic content for each sample at different sampling times (Figure S2) revealed a strong linear correlation between these measured values (y = 0.012x by linear least squares analysis, R^2^ = 0.973; y = 0.008x by linear least squares analysis, R^2^ = 0.999; and y = 0.006x by linear least squares analysis, R^2^ = 0.999 for the CTRL, PEP1, and PEP2 samples, respectively). Interestingly, it is not surprising that the antioxidant activity was directly related to the hydrophilic phenols from the PE (R^2^ = 0.991 and R^2^ = 0.997 for PEP1 and PEP2, respectively) and to the sum of phenols from basil (R^2^ = 0.973, R^2^ = 0.999, and R^2^ = 0.999 for the CTRL, PEP1, and PEP2 samples, respectively). Hence, these results suggested that the antioxidant capacity of the pesto was strongly related to these phenolic fractions. A study carried out by Morellò et al. [72] on the antioxidant activity of phenolic compounds in the olive pulp and olive oil of the Arbequina variety showed that the phenolic compounds with a stronger anti-radical activity were those with two hydroxyl groups in ortho-position to the aromatic ring, such as oleuropein and its derivatives (especially 3,4-DHPEA-EDA), while this activity was lower for the ligstroside derivatives (*p*-HPEA-EDA and *p*-HPEA-EA). Moreover, our results agree with those of previous studies that investigated the phenolic composition and antioxidant activity of basil (*Ocimum basilicum* L.) [69,71]. Jayasinghe et al. [69] observed a high linear correlation between total antioxidant capacity and phenolic content (R^2^ = 0.882), and there was also a positive relationship between antioxidant activity and rosmarinic acid content (R^2^ = 0.839).

### 3.4. Evolution of Volatile Compounds of Pesto

Fresh green pesto is a complex sauce, with each ingredient (e.g., basil leaves, cheese, vegetable oil, pine nuts and/or walnuts, and garlic) contributing to its particular flavour profile [73,74]. Few studies have investigated the volatile fractions of pesto or its ingredients and have reported that the chemical classes of the main flavouring compounds (e.g., alcohols, aldehydes, terpenes, esters, furans, and acids) were similar [2,6,9,18,72,73]. Moreover, the concentration of some volatile compounds varies considerably depending on the genetic and geographical origin, the area of cultivation of each ingredient, technological processes (e.g., pasteurisation or sterilisation), and storage conditions [2,6,9,18,73,74,75].

**Figure 1:**
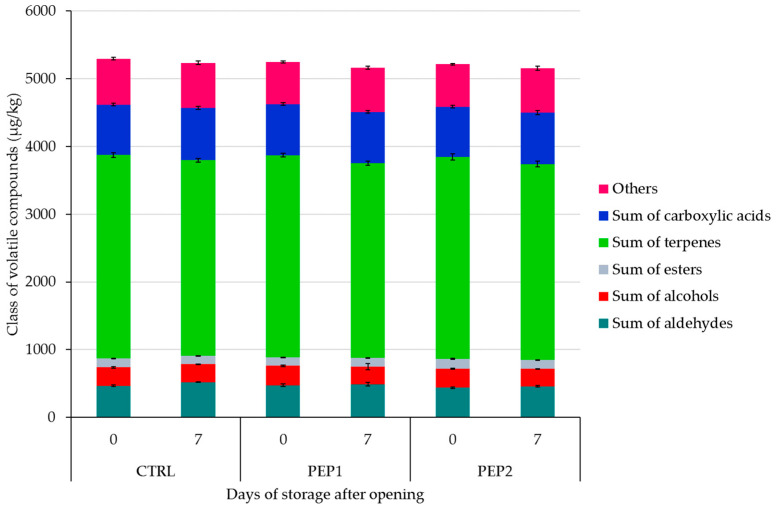
Class of volatile compounds (µg/kg) of the pesto samples at opening (day 0) and at the end of the SSL (7th day of storage after opening). The results are the mean of two independent analytical determinations ± standard deviation. Legend: CTRL, control plus 0.06 g of ascorbic acid/kg of pesto and 1 g of sorbic acid/kg of pesto; PEP1, plus PE corresponding to 250 mg of phenols/kg of pesto; and PEP2, plus PE corresponding to 500 mg of phenols/kg of pesto.

The volatile compounds that characterised the headspace of the pesto samples and their evolution over time are listed in Table S5. In terms of the class of volatile compounds, the pesto formulations did not differ significantly (*p* > 0.05), except for the sum of esters in the PEP2 samples. In terms of the individual classes of volatile compounds (Figure 1), terpenes were the most representative, accounting for 57% and 56% of the total volatile compound content, respectively. In particular, linalool and eucalyptol were the most abundant volatile compounds with values of 790.7 ± 21.8 µg/kg and 649.1 ± 11.5 µg/kg in the CTRL, 781.5± 15 µg/kg and 648.3 ± 11.3 µg/kg in PEP1, and 789.2 ± 19.5 µg/kg and 637.4 ±18.3 µg/kg in PEP2, respectively, followed by β-myrcene, β-ocimene, and α-pinene (Table S5). These compounds, according to the literature, contribute in a non-negligible way to the pesto flavour, which was responsible for the ‘green’, ‘floral’, ‘lemon’, ‘fruity’, ‘slight leafy’, ‘peanutty’, and many other olfactive sensory notes [9,73,74,75]. Comparing these results with those of previous studies on the volatile profile of basil leaves and pesto, similar and often higher values were found [6,9,18,73]. Nicosia et al. [9], who studied the SSL of commercial ‘Pesto alla Genovese’ by simulating household use, observed that terpenes were the main class of volatile compounds in the pesto samples, accounting for approximately 72–83% of the total chromatographic area. Their results partially confirm ours. In addition to the volatile compounds originating from basil leaves and pine nuts, others from the cheese group, such as butanoic acid, were also detected in higher concentrations at the beginning of storage (430.2 ± 15.8 µg/kg, 433.8 ± 19 µg/kg, and 425.3 ± 16.5 µg/kg in the CTRL, PEP1, and PEP2 samples, respectively), followed by hexanoic acid (239.0 ± 9.7 µg/kg, 235.1 ± 2.8 µg/kg, and 233.2 ± 13.8 µg/kg in the CTRL, PEP1, and PEP2 samples, respectively) (Table S5). Our results agree with those of Qian et al. [76], who identified the odour-active compounds in Parmigiano Reggiano cheese through gas chromatography/olfactometry. These authors found that acetic acid, butanoic acid, hexanoic acid, and octanoic acid were the predominant acids in the flavour composition of Parmigiano Reggiano, which are closely associated with the sensory notes ‘lipolyzed’ and ‘cheesy’ [76].

The volatile composition profile of the pesto changed during the storage conditions simulated for loose foods at a deli counter. These variations in the quali-quantitative composition of the headspace of the samples were presumably due to conversion reactions into other volatile compounds and/or depletion losses [9]. During the SSL, all the samples showed a slight decrease in esters, terpenes, C_5_-C_6_ aldehydes, and alcohols associated with the ‘green’, ‘basil-like’, and ‘fruity’ attributes of pesto [74] (Table S5).

**Figure 2:**
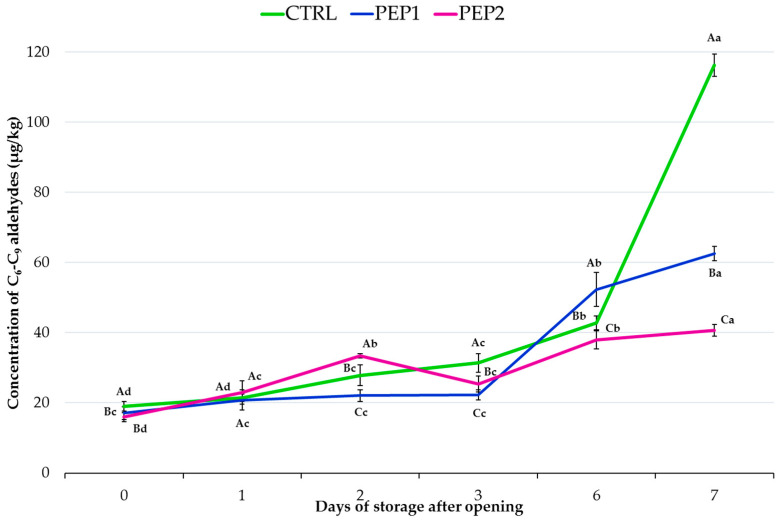
Evolution of the concentrations of C_6_-C_9_ aldehydes (μg/kg) expressed as the sum of hexenal, (E)-2-heptenal, and nonanal aldehydes in the pesto samples during the SSL (at opening (day 0) and 1, 2, 3, 6, and 7 days of storage after opening). The results are the mean of two independent analytical determinations ± standard deviation. Different capital letters (A–C) indicate significant differences among the formulations (*p* < 0.05); different lowercase letters (a–d) indicate significant differences as a function of storage days after opening (*p* < 0.05). Legend: CTRL, control plus 0.06 g of ascorbic acid/kg of pesto and 1 g of sorbic acid/kg of pesto; PEP1, plus PE corresponding to 250 mg of phenols/kg of pesto; and PEP2, plus PE corresponding to 500 mg of phenols/kg of pesto.

Lipid oxidation is considered one of the main causes of the deterioration of pesto during processing, as mentioned above, but also during storage because of the high lipid content [6,9]. This not only leads to nutrient losses but also to the accumulation of secondary lipid oxidation products (e.g., aldehydes and ketones), which are associated with off-flavours and severely limit the shelf life and stability of foods in general and foods sold loose at the deli counter in particular. Therefore, we focused on the evolution of the sum of C_6_-C_9_ aldehydes in the pesto headspace (expressed as the sum of hexanal, *trans*-2-heptenal, and nonanal aldehydes), which are considered suitable markers for measuring the rancidity of EVOOs stored under light exposure [57,58,67]. As discussed for the PV, the trend evaluation of the concentration of C_6_-C_9_ aldehydes varied significantly (*p* < 0.05) among the formulations at the beginning (just after opening) and the end of the storage period (after 7 days of storage after opening) (Figure 2). In particular, the accumulation of C_6_-C_9_ aldehydes produced during the oxidation of linoleic acid over 13-hydroperoxide [77] reached an overall positive variation of 511%, 266%, and 155% for the CTRL, PEP1, and PEP2 samples, respectively. The C_6_-C_9_ aldehydes were up to 5.4 and 3 times higher in the CTRL and PEP1 samples than in the PEP2 ones, respectively. The antioxidant effect of the PE provided a better inhibition of the formation of free radicals than ascorbic acid and the accumulation and subsequent degradation of hydroperoxides, which did not evolve into the neo-formation secondary oxidation products responsible for the ‘rancid’ off-flavour [57,67,77,78,79], but only at the highest dose (PE2). As with the PV and DPPH˙ data, these results clearly indicate that the PE, proportionally to its initial concentration (PEP2 > PEP1), played an important role in counteracting oxidation and, thus, reducing C_6_-C_9_ aldehyde production, while ascorbic acid did not seem to have the same effect. This effect was already evident with the lowest phenol addition (PE1). These results are in agreement with those of Jayasinghe et al. [80], who compared the efficacy of the two plant extracts (Indian gooseberry fruit and sweet basil leaves) rich in flavonoids and phenolic acids and commercial antioxidants (ascorbic acid and α-tocopherol) on the oxidative stability of an oil-in-water emulsion. The results of Jayasinghe et al. [80] showed that ascorbic acid loses its effectiveness, acting as a prooxidant in this emulsion system in accordance with the ‘polar paradox theory’. The mixing of ascorbic acid with other phenolic compounds, on the other hand, could influence the oxidation inhibition reactions in the emulsion system [80].

### 3.5. Changes in the Colour and pH of Pesto

The characteristic green colour of pesto sauce is an important quality attribute for industrial processing and the end product, strongly influencing consumer choice and preferences [2,4,81]. During the processing and storage of pesto, the oxidation of phenols, degradation of chlorophyll, and enzymatic and non-enzymatic browning may occur [4]. These negative colour changes can reduce the intensity of the green colour and, thus, the attractiveness of the product [6]. The colour of the pesto was measured considering the three parameters L*, a*, and b* of the CIE-Lab system, expressing ‘lightness’, ‘green–red’, and ‘blue–yellow’, respectively. Two other parameters, ‘chroma’ (C*) and ‘hue angle’ (*h*), were also evaluated [82]. The results of all the pesto samples after 0, 3, and 7 days of storage after opening are detailed in Table 5. Significant differences (*p* < 0.05) existed among the pesto samples at each sampling time for all colour coordinate score values (L*, a*, b*, C*, and *h*). The values for lightness (L*) showed a narrow range from 86.77 ± 0.01 to 87.55 ± 0.00, with the highest and lowest values recorded for the CTRL and PEP1 samples, respectively. After 7 days of storage after opening, the L* value decreased significantly (*p* < 0.05) for the CTRL and PEP1 samples, except for the PEP2 ones, which showed an opposite trend (Table 5). The slight decrease in L* values suggested that a colour darkened during the SSL due to the appearance of yellow pigments, while the maintenance of the h* value could depend on the decolourisation associated with ageing. Our data were higher than the values found in the literature [6,9,83]. Nicosia et al. [9], who evaluated the CIE-Lab parameters of 20 just-opened commercial pesto samples during the SSL by simulating two scenarios of domestic use and storage, reported that the range of the L* parameter was between 44.34 ± 0.59 and 47.98 ± 0.62 [9]. The wide variability in pesto sauce colour parameters could be attributed to the cultivar [2], agronomic and industrial processing factors, such as original, non-heat-treated raw pesto [4,16], conventional stabilisation techniques [14], or mild preservation techniques [18], other than to differences in the formulation and ratio among ingredients. Since the green colour of pesto is one of the most important features reflecting the final quality of a thermally treated vegetable, Zeppa and Turon [14], and then Turrini et al. [4], proposed the a* parameter as an important quality marker to evaluate the colour change due to chlorophyll degradation. During the simulation of the deli counter storage of fresh pesto, the a* values varied significantly (*p* < 0.05) for all the samples. The lowest a* values were found in the CTRL samples at each sampling time, resulting in a level of higher green intensity than in the PE-enriched products. During the SSL, the a* values slightly decreased in the CTRL and PEP1 samples, while PEP2 showed an opposite trend (i.e., a slight reduction in greenness). Our results are in partial agreement with those reported in other studies on the shelf life of pesto sauces using the accelerated testing approach [6], or simulating real opening and household storage conditions [9]. The decrease in the a* parameter was already observed by Nicosia et al. [9], who found that this value slightly decreased in industrial pesto samples at the beginning of household storage to stabilise lather. An increase in the storage temperature led to a change in colour due to the degradation of chlorophylls, which are particularly sensitive to degradation reactions [4,6]. Moreover, our results are consistent with those of a previous study by Turrini et al. [4], who monitored the colour of basil-based semi-finished products during three months of storage at refrigeration temperature and also evaluated the use of different food additives (ascorbic acid, citric acid, or a mixture of both). These authors concluded that the combined effect of blast chilling and ascorbic acid addition was the best method to maintain the colour of semi-finished basil-based products during storage. At 7 days of storage after opening, the values of the colour coordinates of b*, representing the yellow–blue axis, changed significantly (*p* < 0.05), depending on both the formulations and the storage time, with the highest value being found for the CTRL pesto. This change could indicate a shift towards a brown colour due to the large surface area of the sample in direct contact with oxygen, which favours enzymatic or non-enzymatic browning and accelerates deterioration. In order to delay the quality loss of freshly cut green beans, Lucera et al. [84], testing different packaging systems (unpackaged, microperforated, and non-perforated films), supposed that polyphenoloxidase and peroxidase activity produce quinones, which ultimately cause the browning of fresh-cut fruits and vegetables through polymerisation. However, Zardetto and Barbanti [6] assumed that non-enzymatic browning depends on the reaction between oxidised lipids and proteins. Our finding is consistent with the higher value of the browning index (BI) after 7 days in the CTRL sample (79.06 ± 4.12) than those calculated for the PEP1 (74.62 ± 1.57) and PEP2 samples (64.63 ± 0.27) (Table 5). Interestingly, the presence and the level of addition of PE to the oil phase of the pesto seem to have a positive influence on limiting the browning process, which is more effective than that of ascorbic acid. On the other hand, many authors have demonstrated that ascorbic acid is more effective as an antioxidant than as an enzyme inhibitor [85]. At the beginning of storage (day 0), the colour differences among the tested pesto samples were not easily perceptible to the human eye, as the value of the overall colour variation (ΔE) was low (>2), according to Martínez et al. [86] (Table S6). Except for PEP1, an increase in the ΔE value was observed for the CTRL and PEP2 samples, reaching values of 2.5 and 3.2, respectively, after 7 days of storage after opening (Table 5). Furthermore, the highest ΔE variation was calculated for the CTRL vs. PEP2 samples (6.0) at 7 days (Table S6).

The pH values did not vary significantly (*p* < 0.05) depending on the formulation and storage time, and ranged from 5.26 to 5.37 for the CTRL and PEP2 samples, respectively (Table 6). Similar results were reported by Zardetto and Barbanti [6] for eight commercially available, shelf-stable pesto sauces, with an initial pH of between 4.00 and 5.64. After 7 days of storage after opening, the pH values were close to the initial value (a decrease of 0.03 and 0.06 units for the CTRL and PEP2 samples, respectively), confirming the intrinsic stability of the products.

### 3.6. Sensory Analysis

**Figure 3:**
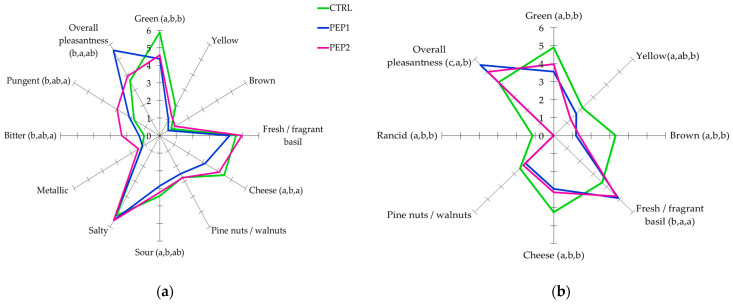
Sensory profile of pesto samples during the SSL: (**a**) spider plot of the sensory data of the pesto samples at opening (day 0); (**b**) spider plot of the sensory data of the pesto samples after 7 days of storage after opening. The results are the mean of the sensory evaluation data. Attributes for which the score values differ significantly (*p* < 0.05) are labelled with different letters between brackets, each referring to samples CTRL, PEP1 and PEP2. Legend: CTRL, control plus 0.06 g of ascorbic acid/kg of pesto and 1 g of sorbic acid/kg of pesto; PEP1, plus PE corresponding to 250 mg of phenols/kg of pesto; and PEP2, plus PE corresponding to 500 mg of phenols/kg of pesto.

Sensory quality is one of the decisive factors for consumer choice after production and during storage. To assess the acceptability of the pesto samples enriched with the PE compared to the CTRL ones, a sensory evaluation was carried out. This evaluation is a supplement to the determinations of the legally required microbiological parameters [52] and chemical properties. The data on appearance, odour, texture, taste, and overall acceptability are graphically illustrated in Figure 3a,b. At the beginning of the experiment (Figure 3a), the addition of PE1 and PE2 to the oily phase of the pesto only affected some sensory attributes in the PEP1 and PEP2 samples studied compared to the CTRL sample, although this effect was dose dependent. The spider plot of the sensory profiles highlights that the PEP2 sample was characterised by a higher intensity of the sensory notes of ‘basil smell’, ‘bitter’, and ‘pungent’ than for the PEP1 and CTRL samples. Since the ‘bitter’ and ‘pungent’ attributes are associated with the presence of oleuropein (3,4-DHPEA-EDA) and ligstroside derivatives (*p*-HPEA-EDA), respectively [30], this result is not surprising. On the other hand, the intensity of these sensory notes was significantly (*p* < 0.05) lower for the PEP1 sample and completely absent for the CTRL sample, and it was more pronounced in the PEP2 sample, which, as mentioned above, had a higher content of these compounds. Instead, the CTRL and PEP1 samples showed higher values for the attributes of ‘cheese’ and ‘pine nut’ related to different volatile compounds such as esters and terpenes (α-pinene, β-pinene, and limonene) [73]. Furthermore, the sensory profile of the CTRL pesto showed significant differences (*p* < 0.05) for the attribute ‘green’. This result was confirmed with the negative values of the a* parameter of the CIE-Lab colour system, indicating ‘greenish’ [4]. The taste attributes of ‘salty’, ‘sour’, and ‘metallic’ were interchangeable in all the samples tested, and none of the samples showed off-flavours. Finally, the PEP1 sample was more appreciated, followed by the PEP2 and CTRL samples (Figure 3a).

Fresh pesto is particularly popular among consumers because of its bright green colour, distinct flavour, texture, and taste [73]. However, these characteristics decrease during the product’s shelf life and may become unacceptable to consumers when compared to fresh products [9]. To investigate this aspect, the sensory analysis was repeated after 7 days of storage after opening, although the evaluation was limited to the appearance and olfactory characteristics because, as the results of the microbiological analyses were not available in real time, the safety of the samples was not guaranteed [87]. The most noticeable changes in the sensory profile of the CTRL sample were a significant (*p* < 0.05) increase in ‘brown’ and ‘yellow’ colours, a decrease in ‘fresh/fragrant basil’, and the development of ‘rancid’, which had a negative impact on the attribute ‘overall pleasantness’ (Figure 3b). These results are in line with the instrumental colour change in the CIE-Lab colorimetric coordinates, the analyses of the chemical oxidation state (in particular, the PV), and the highest accumulation of C_6_-C_9_ aldehydes (hexanal, *trans*-2-heptenal, and nonanal aldehydes) found in the headspaces of the CTRL pesto during the SSL. In addition, the sensory data confirmed that the PEP1 and PEP2 samples were characterised by the highest score for all the attributes studied, especially for those of ‘fresh/fragrant basil’ and ‘overall pleasantness’. These results are consistent with the results of the analysis of the headspace of the pesto samples enriched with the PE, which revealed a higher concentration of α-pinene and β-pinene related to the ‘basil’ and ‘pine nuts’ sensory notes [73].

Although the ‘bitter’ and ‘pungent’ attributes associated with the presence of phenols for the OVW were perceived by the panellists, they did not have a negative impact on the organoleptic properties of the products. Contrarily, PE addition resulted in the absence of the ‘rancid’ off-flavour, which was more pronounced in the CTRL sample, after 7 days of storage after opening.

### 3.7. Multivariate Analysis

**Figure 4:**
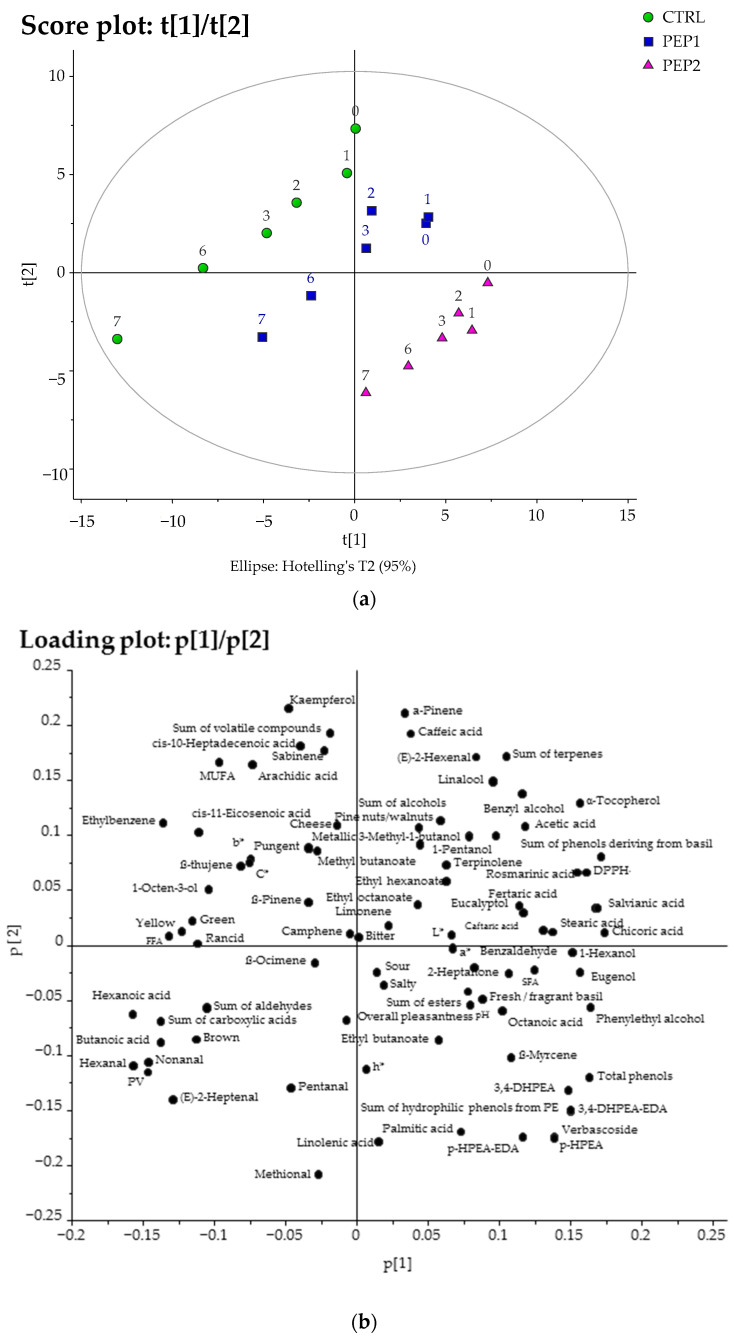
Score (**a**) and loading (**b**) plot of the first two principal components of the PCA model.

**Figure 5:**
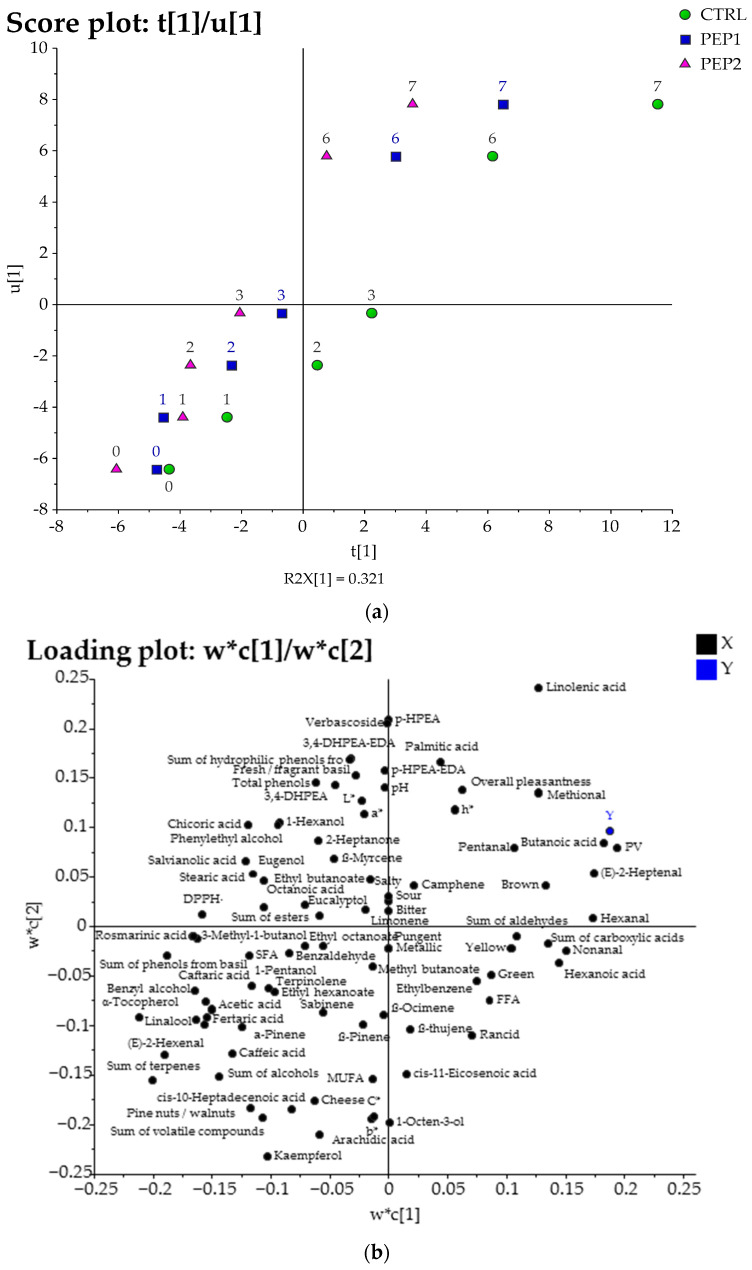
Score (**a**) and loading (**b**) plot of the PLS model of time of storage (Y) vs. analytical and sensory variables (X).

To better understand which chemical compounds, parameters, and sensory attributes were the most relevant for understanding the effects of the addition of the PE on the SSL of the experimental pesto samples, the instrumental and sensory analysis data were subjected to two different multivariate analyses, i.e., principal component analysis (PCA) and partial least squares projections to latent structures (PLS) (Figure 4 and Figure 5, respectively). PCA is widely applied as an unsupervised classification method to interpret the relationships between variables in multivariate datasets [88]. This method aims to assess whether clustering exists within a dataset without using predefined target variables [88]. The whole dataset of the results obtained from the instrumental and sensory analyses of the experimental pesto samples during the SSL (just opened (day 0) and 1, 2, 3, 6, and 7 days of storage after opening) was first elaborated by building a PCA (Figure 4), which showed a clear discrimination of the samples according to their formulations and storage time along PC1 (from left to right of the score, Figure 4a) together with PC2 (from top to bottom of the score, Figure 4a). Subsequently, a PLS (Figure 5) of latent variable analysis was carried out to observe a possible correlation between the evolution of the quality of the pesto samples (CTRL, PEP1, and PEP2) and the storage time after opening. In particular, the PCA model explained 63% of the total variance of the data (with three principal components (PCs)—36% for PC1, 17% for PC2, and 10% for PC3). In the score plot of the first two PCs (Figure 4a), the samples were clearly clustered according to their different formulations together with their storage time. The CTRL samples were furthest to the left side of PC1 and at the top side of PC2, with the PEP1 samples (with a lower PE addition) in between, while the PEP2 samples (with a higher PE addition) were on the opposite side of the CTRL ones. In terms of the days of storage after opening, all the samples of each formulation were furthest to the right side of PC1 at the beginning of the storage simulation and shifted to the left side of PC1 with increasing storage time, except for the PEP2 samples, which were located on the right side of PC1. In addition, the CTRL samples were more elongated than those from PEP2, suggesting that the former underwent greater changes over time. The corresponding loading plot (Figure 4b) showed that the variables most responsible for the distribution of PEP2 samples, followed by PEP1 and CTRL at the beginning of the SSL on the right side of the PC1, were: antioxidant compounds, such as α-tocopherol, phenols from both basil and PE, and DPPH˙ and some terpenes (eugenol, eucalyptol, and β-myrcene); these chemical compounds and parameters are associated with freshness, sensory, and healthy food quality. The PVs, aldehydes C_6_-C_9_ (hexanal, nonanal, *trans*-2-heptenal aldehydes), and sensory attributes, such as ‘yellow’, ‘green’, ‘brown’, and ‘rancid’ off-flavour, were the highest loading variables for the distribution of the CTRL samples and the PEP1 samples at the end of the storage period (after 6 and 7 days of storage after opening, respectively) on the opposite side of the loading plot (left side of PC1) (Figure 4b). On the top side of PC2, kaempferol, α-pinene, the sum of volatile compounds, caffeic acid, sabinene, *trans*-2-hexenal, and the sum of terpenes were the variables largely responsible for the distribution of the pesto samples at the beginning of the days of post-opening; on the bottom side of PC2, the highest loading variables for the pesto samples enriched with the PE were methional, linolenic, and palmitic acids, as well as the hydrophilic phenols from the PE (Figure 4b).

In order to better illustrate the evolution that the pesto samples underwent during the SSL, a PLS model was built in which the days of storage after opening were assigned to the Y variable (Figure 5). With three latent variables, the obtained model explained 98% of the variance of the Y variable (80%, 15%, and 3% for the latent variables, respectively) and 60% of the X variable. In the score plot of t [1]/u [1], the horizontal separation for each sample increased as time progressed (Figure 5a). In particular, the first latent variable showed a narrow distribution of the CTRL samples, which were on the left side of PC1, and the pesto samples, which were enriched with the PE, were on the right, in line with the concentration of PE (Figure 5a). The pesto samples were broadly distributed according to their days of storage after opening, with the pesto samples after 6 and 7 days of storage after opening occupying the upper part of the score plot and the samples stored 0–3 days after opening occupying the lower part. In the relative loading plot (Figure 5b), the parameters and variables positively correlated with Y (time) were those related to primary and secondary oxidation, such as the PV [57,58] and volatile compounds responsible for the unpleasant sensory note (butanoic acid, hexanoic acid, and *trans*-2-heptenal, hexanal, and nonanal aldehydes) [64] and the sensory attribute of ‘brown’, which were located to the right of the first latent variable. In a previous study, Esposto et al. [57], using PLS analyses, observed high correlations between the PVs and volatile compounds, generally recognised as being responsible for EVOO off-flavours [67], and the time of EVOO storage and light exposure. In contrast, α-tocopherol [65,66], the sum of terpenes [73], salvianic acid, and the sum of phenols from basil [2,43], linalool, and *trans*-2-hexenal [73] were quite far from Y and showed a negative correlation with storage time.

## 4. Conclusions

In this work, an analysis of the influence of the PE from OVW as a new natural origin ingredient on the SSL of a fresh pesto was evaluated, simulating the exposure conditions of a product served loose at the deli counter and comparing the results with that obtained using ascorbic acid. The addition of the PE in two different concentrations to the oily phase of the fresh pesto formulation effectively counteracted the lipid oxidation reaction and the formation and accumulation of the volatile compounds responsible for the unpleasant off-flavours in a way that was proportional to the amount of PE used for most parameters. In the CTRL sample, the PV values and C_6_-C_9_ aldehyde contents were higher than in the PEP1 and PEP2 samples (more than 6% and 23% for the first parameter and 46% and 65% for the last one, respectively), indicating a consistently low oxidation ratio.

In the fresh pesto sauces, the total phenolic compounds (expressed as the sum of phenols from basil and from OVW) progressively decreased during the SSL, but more than 71%, 75%, and 82% of the original number of phenols were still present in the CTRL, PEP1, and PEP2 samples, respectively, after 7 days of storage after opening. Both concentrations of PE (250 and 500 mg of phenols/kg of pesto) demonstrated a higher protective effect than ascorbic acid on the phenols of basil, with only 17% and 30% of the amount of this phenolic fraction remaining in the CTRL sample compared to the PEP1 and PEP2 samples, respectively. Moreover, the pesto enriched with the PE provided the equivalent amount of the phenolic species of olives referred to in the health claim according to Reg. CE 432/2012 [62]. The PE had stronger anti-radical activity than the most common antioxidants used in the food industry, which quickly lose their effectiveness when exposed to light and air. Furthermore, the addition of the PE did not adversely affect the sensory quality of the pesto, while the CTRL sample was found to be ‘rancid’ at the end of the SSL.

For the food industry, with a view to progressively reducing and/or replacing the use of chemical additives towards the so-called ‘clean label’, the lower level of PE (250 mg/kg) is satisfactorily effective in achieving this, even if the higher level of PE (500 mg/kg), which is more effective, could be applied without causing any sensory negative effects. The benefits of this approach are many.

This study on the potential use of PEs from OVW as a new antioxidant of natural origin has provided us with promising results, both in terms of reducing food waste in households, catering chains, retail, and/or restaurants, as well as in improving the sustainability of the food industry and the competitiveness of the olive oil sector, where waste to be disposed of, such as OVW, can be turned into a precious co-product.

## Figures and Tables

**Table 1:** Evolution of the concentration of phenolic compounds (mg/kg) of the pesto samples during the SSL (at opening (day 0) and 1, 2, 3, 6, and 7 days of storage after opening).

Days of Storage after Opening	0	1	2	3	6	7
Compound/Sample	CTRL					
Salvianic acid *	39.6 ± 0.8 Ba	38.0 ± 1.9 Ba	34.1 ± 0.3 Cb	29.8 ± 1.6 Cc	28.0 ± 0.3 Bcd	25.2 ± 0.3 Bd
Caftaric acid	16.7 ± 0.3 Ba	13.1 ± 0.1 Bb	8.2 ± 0.2 Bc	7.9 ± 0.7 Bc	8.1 ± 0.4 Bc	7.1 ± 0.2 Ac
Fertaric acid	3.0 ± 0.2 Ca	2.7 ± 0.2 Ba	2.1 ± 0.1 Bb	2.0 ± 0.2 Bc	2.7 ± 0.1 Aa	1.6 ± 0.1 Ad
Caffeic acid	84.7 ± 1.5 Ba	85.1 ± 3.8 ABa	84.4 ± 0.5 Ba	83.1 ± 2.1 Aa	81.2 ± 0.5 Aa	75.9 ± 0.5 Bb
Chicoric acid	48.3 ± 1.5 Ba	48.2 ± 4.5 Aab	44.5 ± 0.3 Cb	40.3 ± 1.8 Bc	38.2 ± 0.3 Cc	30.6 ± 0.6 Cd
Rosmarinic acid	169.6 ± 2.4 Aa	168.4 ± 7 Aa	166.7 ± 0.2 Ba	164.6 ± 2.8 Ba	123.9 ± 1 Cb	117.5 ± 1.1 Cc
Kaempferol	2.5 ± 0.2 Aa	2.1 ± 0.4 Aa	2.5 ± 0.2 Aa	2.2 ± 0.1 Aa	2.2 ± 0.1 Aa	2.1 ± 0.2 Aa
Sum of phenols from basil	364.4 ± 3.3 Ba	357.6 ± 9.4 Aa	342.5 ± 0.7 Cb	329.8 ± 4.3 Cc	284.3 ± 1.2 Cd	259.8 ± 1.4 Ce
Hydroxytyrosol (3,4-DHPEA)	n.d.	n.d.	n.d.	n.d.	n.d.	n.d.
Tyrosol (*p*-HPEA)	n.d.	n.d.	n.d.	n.d.	n.d.	n.d.
Verbascoside	n.d.	n.d.	n.d.	n.d.	n.d.	n.d.
Oleacein (3,4-DHPEA-EDA)	n.d.	n.d.	n.d.	n.d.	n.d.	n.d.
Oleochantal (*p*-HPEA-EDA)	n.d.	n.d.	n.d.	n.d.	n.d.	n.d.
Sum of hydrophilic phenols from PE	n.d.	n.d.	n.d.	n.d.	n.d.	n.d.
Total phenols	364.4 ± 3.3 Ca	357.6 ± 9.4 Ca	342.5 ± 0.7 Cb	329.8 ± 4.3 Cc	284.3 ± 1.2 Cd	259.8 ± 1.4 Ce
	PEP1					
Salvianic acid	41.2 ± 0.8 Aa	42.1 ± 0.6 Aa	36.8 ± 0.4 Bb	34.0 ± 0.8 Bc	33.4 ± 1.6 Ac	33.4 ± 1.0 Ac
Caftaric acid	11.9 ± 0.2 Ca	12.5 ± 0.4 Ba	7.4 ± 0.2 Cb	6.2 ± 0.2 Cc	6.5 ± 0.2 Cc	5.6 ± 0.2 Ac
Fertaric acid	2.3 ± 0.2 Ba	2.7 ± 0.1 Ba	2.0 ± 0.1 Bb	1.6 ± 0.1 Bc	1.6 ± 0.2 Cc	1.4 ± 0.2 Ac
Caffeic acid	92.4 ± 1.4 Aa	87.7 ± 1.1 Aa	93.1 ± 0.1 Aa	80.6 ± 3.8 Ab	80.6 ± 0.3 Ab	80.3 ± 1.7 Ab
Chicoric acid	50.1 ± 0.4 ABab	50.4 ± 0.2 Aa	49.8 ± 0.3 Bab	49.2 ± 0.2 Abc	48.5 ± 0.4 Bc	45.3 ± 0.8 Bd
Rosmarinic acid	162.6 ± 1.8 Ba	163.0 ± 0.2 Aa	162.2 ± 1.1 Ca	160.8 ± 4.8 Ca	141.5 ± 0.8 Bb	135.4 ± 2.1 Bc
Kaempferol	2.2 ± 0.2 Aa	2.1 ± 0.1 Aa	2.3 ± 0.1 Aa	2.0 ± 0.3 Aa	2.0 ± 0.1 Aa	2.0 ± 0.2 Aa
Sum of phenols from basil	362.9 ± 2.4 Ba	360.5 ± 1.3 Aab	353.7 ± 1.2 Bb	334.4 ± 6.1 Bc	314 ± 1.9 Bd	303.5 ± 3 Be
Hydroxytyrosol (3,4-DHPEA)	43.6 ± 2.5 Bb	45.7 ± 0.8 Bab	47.1 ± 0.6 Ba	33.3 ± 0.4 Bc	26.1 ± 0.2 Bd	35.5 ± 0.6 Bc
Tyrosol (*p*-HPEA)	4.1 ± 0.1 Ba	4.1 ± 0.2 Ba	4.0 ± 0.3 Ba	4.0 ± 0.2 Ba	4.1 ± 0.1 Ba	4.1 ± 0.3 Ba
Verbascoside	3.2 ± 0.3 Ba	3.1 ± 0.3 Ba	3.2 ± 0.1 Ba	3.1 ± 0.3 Ba	3.2 ± 0.1 Ba	3.2 ± 0.2 Ba
Oleacein (3,4-DHPEA-EDA)	185.8 ± 0.6 Ba	180.4 ± 2.8 Bab	176.0 ± 4.5 Bbc	168.7 ± 2 Bc	135.8 ± 3.9 Bd	102.7 ± 1.3 Be
Oleochantal (*p*-HPEA-EDA)	n.d.	n.d.	n.d.	n.d.	n.d.	n.d.
Sum of hydrophilic phenols from PE	236.6 ± 2.6 Ba	233.3 ± 3 Ba	230.3 ± 4.6 Ba	209.2 ± 2.1 Bb	169.1 ± 3.9 Bc	145.5 ± 1.5 Bd
Total phenols	599.5 ± 3.6 Ba	593.8 ± 3.2 Bab	584 ± 4.8 Bb	543.5 ± 6.5 Bc	483.1 ± 4.4 Bd	449.0 ± 3.3 Be
	PEP2					
Salvianic acid	42.0 ± 0.7 Aa	41.4 ± 0.7 Aa	42.0 ± 0.6 Aa	39.8 ± 0.6 Aa	32.5 ± 0.4 Ab	35.3 ± 2.0 Ab
Caftaric acid	21.4 ± 1 Aab	22 ± 1.4 Aa	18.8 ± 0.3 Abc	18 ± 0.5 Ac	9.4 ± 0.1 Ad	7.2 ± 2.6 Ae
Fertaric acid	4.2 ± 0.2 Aa	4.1 ± 0.3 Aa	3.2 ± 0.1 Ab	3.2 ± 0.2 Ab	2.1 ± 0.1 Bc	1.6 ± 0.4 Ac
Caffeic acid	79.3 ± 1.6 Ca	79.1 ± 4.1 Ba	79.5 ± 0.7 Ca	80.9 ± 1.1 Aa	79.0 ± 1.7 Aa	78.1 ± 0.3 Aa
Chicoric acid	51.9 ± 0.9 Aab	52.4 ± 2.1 Aa	51.4 ± 0.7 Aab	50.7 ± 0.7 Aab	50.5 ± 0.3 Aab	49.2 ± 0.5 Ab
Rosmarinic acid	172.5 ± 1.7 Aa	171.0 ± 5.5 Aab	170 ± 0.3 Aab	168.9 ± 0.2 Aab	169.1 ± 1.3 Aab	165 ± 1.1 Ab
Kaempferol	2.1 ± 0.2 Aa	2.0 ± 0.3 Aa	1.9 ± 0.1 Ba	2.0 ± 0.3 Aa	1.9 ± 0.1 Aa	1.9 ± 0.1 Aa
Sum of phenols from basil	373.3 ± 2.8 Aa	371.9 ± 7.3 Aab	366.8 ± 1.2 Aab	363.6 ± 1.6 Ab	344.5 ± 2.2 Ac	338.2 ± 3.6 Ac
Hydroxytyrosol (3,4-DHPEA)	76.0 ± 1.1 Aa	73.9 ± 0.7 Aa	75.4 ± 0.8 Aa	49.0 ± 0.6 Ac	53.5 ± 1.1 Ab	50.5 ± 0.8 Abc
Tyrosol (*p*-HPEA)	7.5 ± 0.3 Aa	7.6 ± 0.2 Aa	7.7 ± 0.1 Aa	7.7 ± 0.2 Aa	7.6 ± 0.1 Aa	7.5 ± 0.1 Aa
Verbascoside	6.5 ± 0.5 Aa	6.3 ± 0.2 Aa	6.3 ± 0.4 Aa	6.4 ± 0.3 Aa	6.4 ± 0.1 Aa	6.2 ± 0.4 Aa
Oleacein (3,4-DHPEA-EDA)	343.0 ± 4.4 Aa	334.0 ± 4.1 Ab	330.8 ± 2 Abc	324.3 ± 0.7 Ac	306.8 ± 1 Ad	257.9 ± 3.7 Ae
Oleochantal (*p*-HPEA-EDA)	2.4 ± 0.2 Aa	2.3 ± 0.1 Aa	2.2 ± 0.3 Aa	2.3 ± 0.1 Aa	2.3 ± 0.2 Aa	2.2 ± 0.1 Aa
Sum of hydrophilic phenols from PE	435.5 ± 4.6 Aa	424.1 ± 4.1 Ab	422.4 ± 2.2 Ab	389.7 ± 1 Ac	376.5 ± 1.5 Ad	324.3 ± 3.8 Ae
Total phenols	808.8 ± 5.3 Aa	796.0 ± 8.4 Aab	789.2 ± 2.5 Ab	753.3 ± 1.9 Ac	721.1 ± 2.7 Ad	662.5 ± 5.2 Ae

* Results are the mean of two independent analytical determinations ± standard deviation. Different capital letters (A–C) in the row, within each storage day, indicate significant differences among the formulations (*p* < 0.05); different lowercase letters (a–e) in the row, within each formulation, indicate significant differences as a function of storage days after opening (*p* < 0.05). n.d. not detected. Legend: CTRL, control plus 0.06 g of ascorbic acid/kg of pesto and 1 g of sorbic acid/kg of pesto; PEP1, plus PE corresponding to 250 mg of phenols/kg of pesto; and PEP2, plus PE corresponding to 500 mg of phenols/kg pesto.

**Table 2:** Changes in the regulatory quality parameters of oils extracted from pesto samples during the SSL (at opening (day 0) and 1, 2, 3, 6, and 7 days of storage after opening).

Days of Storage after Opening	CTRL	PEP1	PEP2
	Free acidity (%)		
0	0.56 ± 0.02 Ab	0.57 ± 0.05 Aa	0.58 ± 0.02 Aa
1	0.58 ± 0.01 Aab	0.56 ± 0.02 Aa	0.56 ± 0.03 Aa
2	0.60 ± 0.01 Aab	0.56 ± 0.01 Aa	0.55 ± 0.01 Ba
3	0.60 ± 0.02 Aab	0.57 ± 0.01 Aa	0.58 ± 0.02 Aa
6	0.63 ± 0.02 Aa	0.59 ± 0.02 Aa	0.56 ± 0.01 Aa
7	0.60 ± 0.01 Aab	0.57 ± 0.01 Aa	0.57 ± 0.02 Aa
	Peroxide values (meq O_2_/Kg oil)		
0	8.6 ± 0.3 Abc	8.2 ± 0.3 Ad	8.9 ± 0.3 Abc
1	8.7 ± 0.3 Abc	8.8 ± 0.4 Acd	8.7 ± 0.2 Abc
2	8.1 ± 0.3 Ac	7.6 ± 0.2 Ad	7.3 ± 0.1 Ad
3	10.8 ± 0.3 Ab	10.3 ± 0.5 Ac	8.3 ± 0.3 Bc
6	15.4 ± 0.5 Aa	13.9 ± 0.5 Ab	9.2 ± 0.5 Bb
7	16.4 ± 0.4 Aa	15.5 ± 0.4 Aa	12.7 ± 0.2 Ba

The results are the mean of two independent analytical determinations ± standard deviation. Different capital letters (e.g., A and B) in the row within each storage day represent significant differences among the formulations (*p* < 0.05); different lowercase letters (a–d) in the column within each formulation represent significant differences as a function of storage days after opening (*p* < 0.05). Legend: CTRL, control plus 0.06 g of ascorbic acid/kg of pesto and 1 g of sorbic acid/kg of pesto; PEP1, plus PE corresponding to 250 mg of phenols/kg of pesto; and PEP2, plus PE corresponding to 500 mg of phenols/kg of pesto.

**Table 3:** Evolution of the α-tocopherol content (mg/kg) of the oil extracted from the pesto samples during the SSL (at opening (day 0) and 1, 2, 3, 6, and 7 days of storage after opening).

Days of Storage after Opening	CTRL	PEP1	PEP2
	α-Tocopherol content (mg/kg)		
0	438.5 ± 2.2 Aa	439.6 ± 4.7 Aa	439.9 ± 5.1 Aa
1	438.8 ± 3.9 Aa	439.4 ± 4.3 Aa	439.1 ± 4.7 Aa
2	430.0 ± 2.8 Aabc	438.0 ± 0.9 Aa	437.7 ± 1.2 Aa
3	431.6 ± 1.2 Aab	432.1 ± 0.4 Aab	434.1 ± 3.1 Aa
6	424.1 ± 3.9 Abc	427.8 ± 4.8 Aab	432.1 ± 2.0 Aa
7	420.0 ± 2.0 Ac	424.3 ± 0.9 Ab	428.1 ± 3.1 Aa

The results are the mean of two independent analytical determinations ± standard deviation. Different capital letters in the row within each storage day mean significant differences among formulations (*p* < 0.05); different lowercase letters (a–c) in the column within each formulation represent significant differences as a function of storage days after opening (*p* < 0.05). Legend: CTRL, control plus 0.06 g of ascorbic acid/kg of pesto and 1 g of sorbic acid/kg of pesto; PEP1, plus PE corresponding to 250 mg of phenols/kg of pesto; and PEP2, plus PE corresponding to 500 mg of phenols/kg of pesto.

**Table 4:** Evolution of antioxidant activity (µmol TE/g f.w.) of the pesto samples during the SSL (at opening (day 0) and 1, 2, 3, 6, and 7 days of storage after opening).

Days of Storage after Opening	CTRL	PEP1	PEP2
	Antioxidant activity (µmol TE/g f.w.)		
0	4.59 ± 0.08 Aa	4.67 ± 0.04 Aa	4.78 ± 0.1 Aa
1	4.60 ± 0.00 Ca	4.64 ± 0.00 Ba	4.72 ± 0.01 Aa
2	4.12 ± 0.12 Bb	4.60 ± 0.01 Aa	4.75 ± 0.01 Aa
3	4.47 ± 0.01 Ba	4.41 ± 0.12 Bb	4.69 ± 0.01 Aa
6	2.34 ± 0.00 Cc	4.01 ± 0.01 Bc	4.52 ± 0.01 Ab
7	2.14 ± 0.11 Cc	3.81 ± 0.01 Bc	4.17 ± 0.01 Ac

The results are the mean of two independent analytical determinations ± standard deviation. Different capital letters (A–C) in the row within each storage day represent significant differences among the formulations (*p* < 0.05); different lowercase letters (a–c) in the column within each formulation represent significant differences as a function of storage days after opening (*p* < 0.05). Legend: CTRL, control plus 0.06 g of ascorbic acid/kg of pesto and 1 g of sorbic acid/kg of pesto; PEP1, plus PE corresponding to 250 mg of phenols/kg of pesto; and PEP2, plus PE corresponding to 500 mg of phenols/kg of pesto.

**Table 5:** Changes in the CIE-Lab colorimetric coordinates (L*, a*, and b*), chroma (C*), hue angle (*h*), colour variation (ΔE), and browning index (BI) in pesto samples during the SSL (at opening (day 0) and 3 and 7 days of storage after opening).

	L*		
Days of storage after opening	CTRL	PEP1	PEP2
0	87.55 ± 0.00 Aa	86.40 ± 0.01 Ba	86.77 ± 0.01 Ca
3	86.62 ± 0.04 Ab	88.59 ± 0.00 Bb	86.41 ± 0.02 Cb
7	85.36 ± 0.01 Ac	85.90 ± 0.01 Bc	88.70 ± 0.01 Cc
	a*		
Days of storage after opening	CTRL	PEP1	PEP2
0	−12.94 ± 0.01 Aa	−12.34 ± 0.02 Ba	−12.83 ± 0.01 Ca
3	−13.06 ± 0.01 Ab	−12.12 ± 0.00 Bb	−13.03 ± 0.01 Ab
7	−13.41 ± 0.01 Ac	−12.42 ± 0.00 Bc	−12.58 ± 0.00 Cc
	b*		
Days of storage after opening	CTRL	PEP1	PEP2
0	53.06 ± 0.11 Aa	51.49 ± 0.00 Ba	51.69 ± 0.00 Ba
3	52.76 ± 0.02 Ab	49.62 ± 0.00 Bb	52.62 ± 0.05 Ab
7	54.10 ± 0.02 Ac	52.08 ± 0.03 Bc	49.18 ± 0.00 Cc
	C*		
Days of storage after opening	CTRL	PEP1	PEP2
0	54.61 ± 0.10 Aa	52.95 ± 0.00 Ba	53.26 ± 0.01 Ca
3	54.35 ± 0.02 Ab	51.08 ± 0.00 Bb	54.21 ± 0.04 Cb
7	55.74 ± 0.02 Ac	53.54 ± 0.03 Bc	50.74 ± 0.04 Cc
	*h*		
Days of storage after opening	CTRL	PEP1	PEP2
0	103.70 ± 0.03 Aa	103.48 ± 0.02 Ba	103.94 ± 0.02 Ca
3	103.91 ± 0.00 Ab	103.72 ± 0.00 Bb	103.91 ± 0.00 Aa
7	103.92 ± 0.01 Ab	103.41 ± 0.01 Bc	104.36 ± 0.01 Cb
	CTRL	PEP1	PEP2
ΔE	2.47 ± 0.09 B	0.78 ± 0.04 C	3.17 ± 0.01 A
BI	79.06 ± 4.12 B	74.62 ± 1.57 AB	64.63 ± 0.27 B

The results are the mean of two independent analytical determinations ± standard deviation. Different capital letters (A–C) in the row within each storage day represent significant differences among the formulations (*p* < 0.05); different lowercase letters (a–c) in the column within each formulation represent significant differences as a function of storage days after opening (*p* < 0.05). Legend: CTRL, control plus 0.06 g of ascorbic acid/kg of pesto and 1 g of sorbic acid/kg of pesto; PEP1, plus PE corresponding to 250 mg of phenols/kg of pesto; and PEP2, plus PE corresponding to 500 mg of phenols/kg of pesto.

**Table 6:** Evolution of pH in pesto samples during the SSL (at opening (day 0) and 1, 2, 3, 6, and 7 days of storage after opening).

Days of Storage after Opening	CTRL	PEP1	PEP2
0	5.26 ± 0.05 Aa	5.22 ± 0.06 Aa	5.37 ± 0.05 Aa
1	5.25 ± 0.02 Aa	5.38 ± 0.06 Aa	5.33 ± 0.02 Aa
2	5.24 ± 0.03 Aa	5.42 ± 0.11 Aa	5.33 ± 0.04 Aa
3	5.30 ± 0.10 Aa	5.30 ± 0.04 Aa	5.31 ± 0.02 Aa
6	5.26 ± 0.08 Aa	5.35 ± 0.02 Aa	5.32 ± 0.06 Aa
7	5.23 ± 0.07 Aa	5.35 ± 0.02 Aa	5.31 ± 0.04 Aa

The results are the mean of two independent analytical determinations ± standard deviation. Different capital letters in the row, within the individual storage days, indicate significant differences among formulations (*p* < 0.05); the same lowercase letters in the column, within the individual formulations, indicate significant differences depending on the storage days (*p* < 0.05). Legend: CTRL, control plus 0.06 g of ascorbic acid/kg of pesto and 1 g of sorbic acid/kg of pesto; PEP1, plus PE corresponding to 250 mg of phenols/kg of pesto; and PEP2, plus PE corresponding to 500 mg of phenols/kg of pesto.

## Data Availability

All data are contained within the article and supplementary material.

## Supplementary Materials

The following supporting information can be downloaded at: https://www.mdpi.com/article/10.3390/antiox13010128/s1, Figure S1: Evolution of total phenol concentrations (mg/kg) (a), sum of phenols from basil (mg/kg) (b), and sum of hydrophilic phenols from the PE (mg/kg) (c) in the pesto samples during the SSL (at opening (day 0) and 1, 2, 3, 6, and 7 days of storage after opening); Figure S2: Correlation plot between the DPPH˙ values and the total phenol concentration of each pesto sample at different sampling times (just after opening (day 0), and 1, 2, 3, 6, and 7 days of storage after opening); Table S1: The chemical composition of the PE employed for the manufacturing of the experimental pesto; Table S2: Chemical and spectroscopic characteristics of phenolic compounds revealed using UHPLC-DAD-Q-TOF/MS analysis; Table S3: Hydrophilic phenol concentrations (mg/kg) of the phenolic extract (PE) in pesto samples just after opening (day 0); Table S4: Changes in the fatty acid composition of the oil extract from pesto samples over time (just after opening (day 0) and 1, 2, 3, 6, and 7 days of storage after opening). Table S5: Evolution of the volatile compounds (µg/kg) of the pesto samples during the SSL (at opening (day 0) and 1, 2, 3, 6, and 7 days of storage after opening). Table S6: Colour variation (ΔE) in pesto samples during the SSL (at opening (day 0) and 3 and 7 days of storage after opening).
