# Supplementary material for: Evaluation of the Effect of an Olive Phenolic Extract on the Secondary Shelf Life of a Fresh Pesto
Source: Antioxidants (Basel). 2024 Jan 20;13(1):128. doi: 10.3390/antiox13010128 (PMC10813149; doi:10.3390/antiox13010128)
Supplement: Supplementary file 1 [file antioxidants-13-00128-s001.zip › antioxidants-2778495-supplementary.pdf]

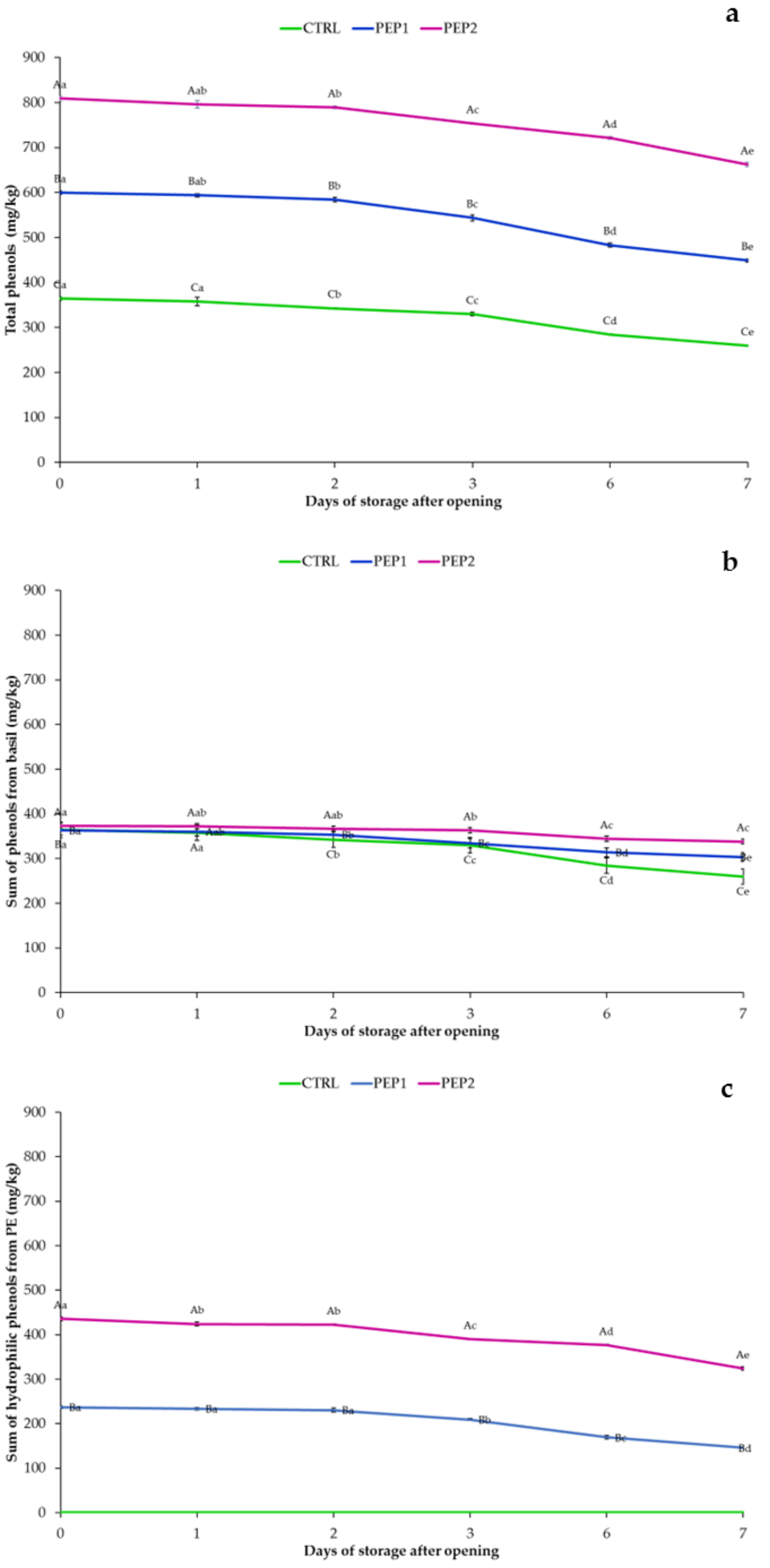

Figure S1. Evolution of total phenol concentrations (mg/kg) (a), sum of phenols from basil (mg/kg) (b), and sum of hydrophilic phenols from the PE (mg/kg) (c) in the pesto samples during the SSL (at opening (day 0) and 1, 2, 3, 6, and 7 days of storage after opening). Results are the mean of two different determinations. The values of sum of phenols from basil (mg/kg) are expressed as the sum of salvianic acid, caftaric acid, fertaric acid, caffeic acid, chicoric acid, rosmarinic acid and kaempferol; The values of sum of hydrophilic phenols from PE (mg/kg) are expressed as the sum

hydroxytyrosol (3,4-DHPEA), tyrosol (*p*-HPEA), verbascodide, oleacein (3,4-DHPEA-EDA) and oleochantal (*p*-HPEA-EDA). Legend: CTRL, control plus 0.06 g ascorbic acid/kg pesto and 1 g sorbic acid/kg pesto; PEP1, plus PE corresponding to 250 mg phenols/kg pesto; and PEP2, plus PE corresponding to 500 mg phenols/kg pesto.

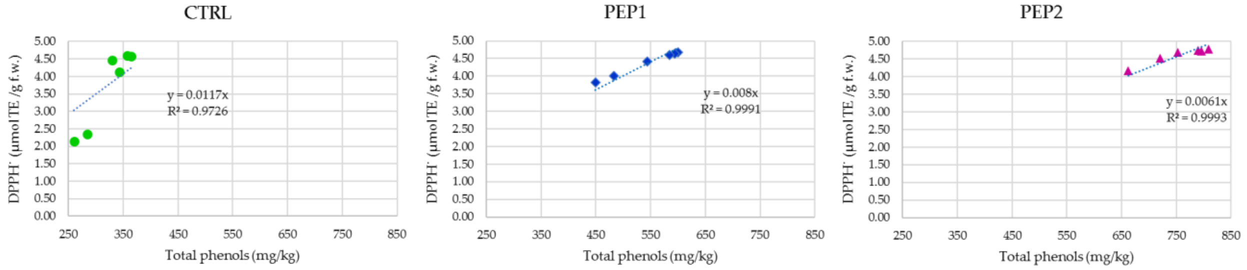

Figure S2. Correlation plot between DPPH˙ values (µmol TE/g f.w.) and total phenol concentration (mg/kg) of each pesto sample at different samplings (just after opening (day 0), and 1, 2, 3, 6, and 7 days of storage after opening). Legend: CTRL, control plus 0.06 g ascorbic acid/kg pesto and 1 g sorbic acid/kg pesto; PEP1, plus PE corresponding to 250 mg phenols/kg pesto; and PEP2, plus PE corresponding to 500 mg phenols/kg pesto.

Table S1. Chemical composition of PE employed for the manufacture of the experimental pesto.

Hydroxytyrosol (3,4-DHPEA) (mg/g)*	80.9 ± 0.3
Tyrosol (*p*-HPEA) (mg/g)	14.6 ± 0.0
Vanillic acid (mg/g)	0.7 ± 0.0
Verbascoside (mg/g)	10.4 ± 0.2
Oleacein (3,4-DHPEA-EDA) (mg/g)	636.0 ± 3.3
Oleochantal (*p*-HPEA-EDA) (mg/g)	5.7 ± 0.1
Total phenols (mg/g)	748.3 ± 3.3
Purity (%)	74.8

* Results are the mean of two different determinations ± standard deviation.

Table S2. Chemical and spectroscopic characteristics of phenolic compounds revealed by UHPLC-DAD-Q-TOF/MS analysis.

Assignment	Molecular formula	retention time (min)	[M-H] Calculated	Expected	Score	Difference (ppm)
Salvianic acid A	$C_9H_{10}O_5$	1.86	197.0455	197.0454	98.86	-0.51
Caftaric acid	$C_{13}H_{12}O_9$	2.24	311.0409	311.041	98.95	0.60
Fertaric acid	$C_{14}H_{14}O_9$	2.9	325.0565	325.0563	98.81	-0.40

Caffeic acid	$C_9H_8O_4$	3.84	179.035	179.0351	99.81	0.83
Chicoric acid	$C_{22}H_{18}O_{12}$	5.77	473.0725	473.0729	99.45	0.84
Salvianolic acid E	$C_{36}H_{30}O_{16}$	6.80	717.1461	717.1456	97.67	-0.65
Rosmarinic acid	$C_{18}H_{16}O_8$	7.28	359.0772	359.0773	99.61	0.38
Salvianolic acid L	$C_{36}H_{30}O_{16}$	7.95	717.1461	717.1455	97.19	-0.93
Salvianolic acid B	$C_{36}H_{30}O_{16}$	10.04	717.1461	717.1451	97.19	-0.98

[41] Lee, J.; Chan, B.L.S.; Mitchell, A.E. Identification/Quantification of Free and Bound Phenolic Acids in Peel and Pulp of Apples (Malus domestica) Using High Resolution Mass Spectrometry (HRMS). *Food Chem.* **2017**, 215, 301–310.

Table S3. Hydrophilic phenols concentration (mg/kg) of phenolic extract (PE) in pesto samples just after opening (day 0).

	CTRL	PEP1	PEP2
Hydroxytyrosol (3,4-DHPEA)*	n.d.	43.6±2.5B	76±1.1A
Tyrosol (*p*-HPEA)	n.d.	4.1±0.1B	7.5±0.3A
Verbascoside	n.d.	3.2±0.3B	6.5±0.5A
Oleacein (3,4-DHPEA-EDA)	n.d.	185.8±0.6B	343±4.4A
Oleochantal (*p*-HPEA-EDA)	n.d.	n.d.	2.4±0.2A
Sum of hydrophilic phenols from PE	n.d.	236.6±2.6B	435.5±4.6A

* Results are the mean of two independent analytical determinations ± standard deviation. Different uppercase letters (A-B) in the row, represent significant differences among the formulations ($p < 0.05$). n.d. not detected. Legend: CTRL, Control plus 0,06 g/kg ascorbic acid and 1 g/kg sorbic acid; PEP1, plus PE equivalent to 250 mg phenols/kg of pesto; and PEP2, plus PE equivalent to 500 mg phenols/kg of pesto.

Table S4. Changes in fatty acid composition of oil extract from pesto samples over time (just after opening (day 0) and 1, 2, 3, 6, and 7 days of storage after opening).

Days of storage after opening	0			1			2			3			6			7		
Compound/Samples	CTRL	PEP1	PEP2	CTRL	PEP1	PEP2	CTRL	PEP1	PEP2	CTRL	PEP1	PEP2	CTRL	PEP1	PEP2	CTRL	PEP1	PEP2
Palmitic acid (C16: 0)	14.1 ±0.2	14.2 ±0.0	14.2 ±0.3	14.1 ±0.2	14.2 ±0.7	14.2 ±0	14.1 ±0.9	14.2 ±0	14.2 ±0.5	14.1 ±0.6	14.2 ±0.4	14.2 ±0.2	14.1 ±0.2	14.2 ±0	14.2 ±0	14.2 ±0	14.2 ±0.1	14.2 ±0
Palmitoleic acid (C16: 1)	0.2± 0.0	0.2± 0.0	0.2± 0.0	0.2± 0.0	0.2± 0.1	0.2± 0.0	0.2± 0.0	0.2± 0.0	0.2± 0.0	0.2± 0.0	0.2± 0.0	0.2± 0.0	0.2± 0.0	0.2± 0.0	0.2± 0.0	0.2± 0.0	0.2± 0.0	0.1± 0.0
Margaric acid (C17: 0)	0.1± 0.0	0.1± 0.0	0.1± 0.0	0.1± 0.0	0.1± 0.1	0.1± 0.0	0.1± 0.0	0.1± 0.0	0.1± 0.0	0.1± 0.0	0.1± 0.0	0.1± 0.0	0.1± 0.0	0.1± 0.0	0.1± 0.0	0.1± 0.0	0.1± 0.0	0.1± 0.0
cis-10-Heptadecenoic acid (C17: 1)	0.1± 0.0	n.d.	n.d.	0.1± 0.0	n.d.	n.d.	0.1± 0.0	n.d.	n.d.	0.1± 0.0	n.d.	n.d.	0±0	n.d.	n.d.	0±0	n.d.	n.d.
Stearic acid (C18: 0)	4.4± 0.0	4.4± 0.0	4.4± 0.0	4.4± 0.0	4.5± 0.2	4.5± 0.2	4.4± 0.1	4.4± 0.0	4.4± 0.0	4.3± 0.1	4.4± 0.1	4.4± 0.1	4.3± 0.0	4.4± 0.0	4.4± 0.0	4.3± 0.0	4.4± 0.0	4.4± 0.0
Oleic acid (C18: 1n9c)	32.7 ±0.1	32.7 ±0.1	32.7 ±0.6	32.7 ±0.1	32.7 ±1.0	32.7 ±0	32.7 ±0.3	32.7 ±0.1	32.7 ±0.7	32.7 ±0.2	32.7 ±0.8	32.7 ±0.3	32.7 ±0.1	32.7 ±0	32.8 ±0	32.7 ±0.1	32.7 ±0.1	32.8 ±0.1
Linoleic acid (C18: 2n6c)	47.3 ±0.1	47.3 ±0.1	47.3 ±0.2	47.3 ±0.1	47.3 ±0.2	47.3 ±0	47.3 ±0.5	47.3 ±0.1	47.3 ±0.2	47.3 ±0.3	47.3 ±0.2	47.3 ±0.1	47.3 ±0.1	47.3 ±0	47.3 ±0.0	47.3 ±0	47.3 ±0	47.2 ±0.0
Linolenic acid (C18: 3n3)	0.7± 0.0	0.7± 0.0	0.7± 0.0	0.7± 0.0	0.7± 0.0	0.7± 0.0	0.7± 0.0	0.7± 0.0	0.7± 0.0	0.7± 0.0	0.7± 0.0	0.8± 0.0	0.7± 0.2	0.7± 0.0	0.8± 0.0	0.7± 0.0	0.8± 0.0	0.8± 0.0
Arachidic acid (C20: 0)	0.3± 0.0	0.3± 0.0	0.2± 0.0	0.3± 0.0	0.2± 0.0	0.2± 0.0	0.3± 0.0	0.3± 0.0	0.3± 0.0	0.3± 0.0	0.3± 0.0	0.2± 0.0	0.3± 0.2	0.2± 0.0	0.2± 0.0	0.3± 0.0	0.2± 0.0	0.2± 0.0
cis-11-Eicosenoic acid (C20: 1n9)	0.2± 0.0	0.1± 0.0	0.1± 0.0	0.2± 0.0	0.1± 0.0	0.1± 0.0	0.2± 0.0	0.2± 0.0	0.2± 0.0	0.2± 0.0	0.1± 0.0	0.1± 0.0	0.2± 0.0	0.1± 0.0	0.1± 0.0	0.2± 0.0	0.2± 0.0	0.1± 0.0
SFA	18.9 ±0.2	18.9 ±0.0	19.0 ±0.3	18.9 ±0.2	19± 0.7	19.0 ±0.0	18.9 ±0.9	18.9 ±0.0	19.0 ±0.5	18.9 ±0.6	18.9 ±0.4	18.9 ±0.2	18.8 ±0.3	18.9 ±0	18.9 ±0.0	18.9 ±0	18.9 ±0.1	18.9 ±0.1
MUFA	33.1 ±0.1	33.1 ±0.1	33.0 ±0.6	33.1 ±0.1	33.1 ±1.0	33.0 ±0.1	33.1 ±0.3	33.1 ±0.1	33.1 ±0.7	33.1 ±0.2	33.1 ±0.8	33.0 ±0.3	33.1 ±0.1	33.1 ±0	33.0 ±0	33.1 ±0	33.1 ±0.1	33.0 ±0.1
PUFA	48.0 ±0.1	48.0 ±0.1	48.0 ±0.2	48.0 ±0.1	48.0 ±0.2	48.0 ±0.0	48.0 ±0.5	48.0 ±0.1	48.0 ±0.2	48.0 ±0.3	48.0 ±0.2	48.0 ±0.1	48.0 ±0.3	48.0 ±0.0	48.0 ±0.0	48.0 ±0	48.0 ±0.0	48.0 ±0.0

Results are the mean of two independent analytical determinations ± standard deviation. There is no significant difference among the formulation ($p < 0.05$) and within each formulation during the days of storage ($p < 0.05$). Legend: CTRL, Control plus 0,06 g/kg ascorbic acid and 1 g/kg sorbic acid; PEP1, plus PE1 equivalent to 250 mg phenols/kg of pesto; and PEP2, plus PE2 equivalent to 500 mg phenols/kg of pesto.

Table S5. Evolution of volatile compounds (μg/kg) of pesto samples during SSL (at opening ((day 0) and 1, 2, 3, 6, and 7 days of storage after opening).

Days of storage after opening	0	1	2	3	6	7
	CTRL					
Aldehydes*						
Pentanal	23.2±1.1Bc	28.9±0.8Bb	27.9±2.6Bb	27.6±2.6Bb	31.6±1.9Ab	37.2±0.3Aa
Hexanal	5.8±0.2Ae	9.3±0.1Ad	14.1±1Ac	15±0.3Ac	19.7±0.2Ab	38.5±1.9Aa
(E)-2-Hexenal	416.8±12.5Aa	389.6±1Bab	384.7±2.8Abc	372.7±4.5Abc	373.1±17.4Abc	360.5±2.4Ac
(E)-2-Heptenal	11.6±0.6Acd	10.5±0.7Ad	12±1.1Bcd	14.6±0.6Ac	20.5±0.3Bb	73.9±2.5Aa
Nonanal	1.7±0.2Ac	1.6±0Ac	1.7±0.2Ac	1.9±0Ac	2.5±0.1Ab	3.8±0.1Aa
Benzaldehyde	8.5±0.4Aa	8.4±0.3Aa	8.3±0.5Aa	8.0±0.4Aa	8.1±0.3Aa	8.3±0.3Aa
Sum of aldehydes	467.5±12.6Aa	448.3±1.4Aa	448.7±4.1Aa	439.6±5.2Aa	455.5±17.6Aa	522.1±4Ab
Alcohols						
3-Methyl-1-butanol	18±0.9Aa	17.5±0Aa	17.6±0Ba	17±1.1Aa	17.5±1.2Aa	17.4±0.1Aa
1-Pentanol	38.2±1.5Bab	38.8±0.8Ba	35.8±0.8Abc	33.6±0.2Bc	38.4±1.2Aa	28.3±0Ad
1-Octen-3-ol	177.8±13.2Aab	172.4±0.1Ab	174.4±1Ab	187.2±4.6Aab	186.4±5.2Aab	194.4±0.5Aa
1-Hexanol	19.1±1Bb	22.4±0.3Ba	14.9±0.2Cc	15.6±0Cc	15.8±0.6Bc	11.5±0.2Bd
Benzyl alcohol	10.7±0.4Aa	10.9±0.2Aa	10±0.4Aa	9.9±0.1Aa	9.9±0.8Aa	8.1±0.2Bb
Phenylethyl alcohol	7±0.3Ba	7.3±0.2Aa	6.5±0.3Bab	6.4±0Cab	6±0.6Cb	5.4±0.2Bb
Sum of alcohols	270.8±13.4Aa	269.3±0.9Aa	259.2±1.3Aa	269.7±4.7Aa	274±5.6Aa	265.1±0.6Aa
Esters						
Methyl butanoate	22.8±1Aa	21.9±0.6ABa	20.3±0.2Ba	22.5±0.4Aa	23.5±2.6Aa	21±1Ba
Ethyl butanoate	48.4±1.5Bc	46.6±0.3Cc	48.5±0.5Cc	66.7±0.5Aa	53.9±2.9Ab	49.6±0.4ABc
Ethyl hexanoate	52.6±2.9Aa	52.4±1.1Aa	54.1±0.3Aa	52.2±0Ba	47±6.4Aa	46.8±1.2Ba
Ethyl octanoate	8.4±0.7Ba	8.7±0.2Aa	8.3±0.4Aa	8.9±0Aa	8.2±0.9Aa	8.4±0.4Aa
Sum of esters	132.2±3.5Aa	129.5±1.4Aa	131.2±0.7Aa	150.4±0.6Ab	132.5±7.5Aa	125.8±1.7Aa
Terpenes						
α-Pinene	276.5±5.6Aa	256.4±1.2Ab	241.5±7.1Abc	226.7±3.3Acd	238±0.7Bbc	216.4±15Ad
Camphene	18.5±0.5Bab	17.7±0.4Bb	19±0.2Ba	18.1±0.3Cab	18.5±0.2Bab	18.8±0.6Aa
β-Pinene	195.7±4.8Aab	184.3±2.5Ac	203.1±1.3Aa	191.1±0.5Ab	203.5±6Aa	194.5±3.3Ab
β-Thujene	118.6±10.2Aab	112.3±2.3Bab	122.4±1.4Bb	124±1.4Aab	122.9±1.3Aab	125.1±1.8Aa
Sabinene	130.2±5.1Aab	131.6±0.1Ba	126.1±3.5Ba	118±0.4Bc	120.2±2.8Abc	115.1±5.4Ac
β-Myrcene	410.6±14.5Aa	411.2±4Ba	401.4±1.4Aa	405±3.8Aa	416.1±45.3Aa	416.9±7.1Aa
Limonene	139.8±5.2Aa	144.5±0.1Aa	132.1±0.5Aab	113.6±2.2Bc	117.7±11.7Bc	121.2±1.3ABbc
Eucalyptol	649.1±11.5Aa	657.4±3.9Aa	627.3±5.9Ab	626.5±9.1Ab	605.6±22.3Bbc	610.3±5.3Ac
β-Ocimene	248±9.9Bb	257.2±1.3Bb	233.7±2Ac	273.5±6Aab	267.4±14.2ABa	282.2±0.8Aa
Terpinolene	24.6±1.5Abc	20.4±1.7Bd	28.3±1.3Aa	27.6±0.3Bab	24.1±1.4Ac	19.3±0.4Ad

Linalool	790.7±21.8Aa	773.9±3.9Aa	773.3±22.8Aa	782±5.2Aa	769.2±17.6Aa	763.6±16.7Aa
Sum of terpenes	3002.3±33.6Aa	2966.9±8.1Aa	2908.2±25.1Aa	2906±13.3Aa	2903.1±56.9Aa	2883.6±25Aa
Carboxylic acids						
Acetic acid	54±2.6Aab	53.3±4.4Bab	55±2Cab	59.5±4.4Aa	57.4±2.3Aa	48.1±3.3Ab
Butanoic acid	430.2±15.8Aa	430.1±18.6Aa	432.5±16.7Aa	439.1±11.1Aa	439.7±21.9Aa	444.8±10.3Aa
Hexanoic acid	239±9.7Aa	242.1±4.4Aa	237.2±4.1Aa	244.6±9.6Aa	250.4±0.2Aa	258.1±20.7Aa
Octanoic acid	20.6±1.5Bab	19.2±1.9Bb	22.2±2Aab	22.3±1.9Aab	23.8±1.1ABa	19.8±1.2Aab
Sum of carboxylic acids	743.9±18.8Aa	744.7±19.7Aa	746.9±17.4Aa	765.5±15.4Aa	771.3±22Aa	770.7±23.4Aa
Others						
2-Heptanone	19.3±1.3Bb	17.5±0.6Bcd	21.1±0.4Bab	18.7±0.6Abc	23.1±1.7Aa	15.1±0.7Bd
Ethylbenzene	493.9±18.3Aa	490.3±9.5Aa	494.9±16.1Aa	495.2±10.7Aa	484.3±8.3Aa	490.1±22.2Aa
Methional	5.2±0.2Ab	4.3±0.6Ab	6.0±0.8Aab	6.2±0.7Aab	6.7±0Aa	7.0±0.5Aa
Eugenol	160±9.5Aa	162.1±12.3Aa	157.1±8Aa	159.3±10.1Ba	152.6±8.8Ba	154.9±7.9Aa
Sum of volatile compounds	5295.1±47.5Aa	5232.7±26.4Aa	5173.2±35.7Aa	5210.6±26.1Aa	5203±65.4Aa	5234.4±41.8Aa
			PEP1			
Aldehydes						
Pentanal	26.5±1.6Ac	36.7±2.7Aa	17.9±1.2Cd	20.2±1.2Cd	26.5±0.1Bc	30.7±1.4Bb
Hexanal	5.2±0.2Bd	9.0±0.7Ac	9.9±0.2Bc	9.9±0.2Bc	19.5±0.5Ab	20.8±0.7Ba
(E)-2-Hexenal	420.5±19.1Aa	406.8±1.5Aa	391.4±15.3Aa	397.6±12.8Aa	391.9±4.1Aa	391.4±30.1Aa
(E)-2-Heptenal	10.1±0.9Ac	10.3±0.7Ac	10.6±1.0Bc	10.6±0.8Bc	31±4.9Ab	39.1±1.2Ba
Nonanal	1.8±0.1Ab	1.5±0.1Ab	1.6±0.2Ab	1.7±0.1Ab	1.8±0.3Bb	2.6±0.6Ba
Benzaldehyde	8.8±0.4Aa	8.0±0.3Aab	7.8±0.4Aabc	7.8±0.1Babc	7.3±0.0Bbc	7.0±0.6Bc
Sum of aldehydes	472.8±19.1Aa	472.3±3.3Ba	439.1±15.4Aa	447.9±12.9Aa	478±6.4Aa	491.6±30.2ABa
Alcohols						
3-Methyl-1-butanol	17±0.4Aa	18.6±3.2Aa	18.2±0.3Aa	18.1±0.6Aa	18.4±0.1Aa	17.3±1.2Aa
1-Pentanol	56.2±1.3Aa	45.2±0.5Ab	31.6±0.7Bd	35.7±0.6Ad	39.1±0.6Ac	33.9±5Ad
1-Octen-3-ol	175.1±12Aa	177.7±3.9Aa	176.9±16.4Aa	176.8±14.9Aa	171.2±9.3ABa	171.2±45.2Aa
1-Hexanol	22.6±0.5Aab	24.4±1.0Aa	18.7±1.4Bb	22.7±1.1Aab	22.1±0.6Aab	20.3±1.8Ab
Benzyl alcohol	10.5±0.5Aa	10.1±0.0Aa	9.7±0.6Aab	9±0.4Aab	8.7±1.3Aab	8.2±0.3Bb
Phenylethyl alcohol	8.4±0.4Aa	7.7±0.1Aab	6.4±0.4Bb	7.2±0.1Bab	7.0±0.1Bb	7.5±1Aab
Sum of alcohols	289.7±12.1Aa	283.7±5.2Aa	261.4±16.5Aa	269.6±15Aa	266.5±9.4Aa	258.2±45.5Aa
Esters						
Methyl butanoate	21.3±1.1Aa	22.6±0.5Aa	22.5±0.4Aa	22.4±1.5Aa	21.1±0.3Aa	23.3±0.7Aa
Ethyl butanoate	40.2±0.9Cd	50.1±2.2Bb	50.8±3.6Bb	58.8±3.7Ba	46.3±2.1Bc	42.1±4.2Bd
Ethyl hexanoate	53.6±2.9Aa	50.3±4.8Aa	50.3±2.7Aa	50±2.4Ba	52.7±3.7Aa	55.4±2.3Aa
Ethyl octanoate	8.1±0.5Ba	9.4±0.7Aa	10.0±1.4Aa	9.1±0.3Aa	9.4±0.2Aa	8.9±0.7Aa
Sum of esters	123.2±3.3Aa	132.3±5.3Aa	133.5±4.7Aa	140.2±4.7Aa	129.5±4.3Aa	129.6±4.9Aa

Terpenes						
α-Pinene	259±3.2Ba	243±2.3Ba	245.9±3.3Aa	247.9±15.3Aa	249.4±2.7Aa	234.5±11.3Aa
Camphene	19.6±0.2Ab	20.5±0.1Ab	18.9±0.6Bb	22.8±0.4Aa	19.5±0.1Ab	20.5±1.5Ab
β-Pinene	191.4±3.1Aa	147±2.6Bd	192.3±4.5Aa	187.7±6.4Aa	168.4±7.2Cb	153.5±4.5Bc
β-Thujene	113.9±1.6Ac	124.9±0.2Abc	132.4±1.4Aab	126.3±3.8Abc	126.4±5.5Abc	140.3±9.6Aa
Sabinene	108.8±1.9Cc	140.1±0.5Aa	133.1±5.5Aab	138.5±0.5Aab	120.8±6.4Ab	119±11.1Abc
β-Myrcene	447.7±18.2Aa	417.2±22.8Ba	419.4±39.5Aa	442.1±29.2Aa	444.7±14.2Aa	416.5±4.5Aa
Limonene	126.6±4.4ABab	136±3.3Ba	91.1±7.9Be	101.2±6.9Cab	118.4±3.5Bbc	116.5±8Bcd
Eucalyptol	648.3±11.3Aa	647.7±9.2Aa	623.4±26.1Aab	619.1±20.6Aab	603.7±8.7Bb	622.2±10.9Aab
β-Ocimene	261.8±5.8ABa	261.6±8.8ABa	260.7±24.7Aa	263.2±19.1Aa	281.7±10.7Aa	272.9±6.2Aa
Terpinolene	27.3±1.7Ab	25.2±0.8Abc	23±1.5Bc	31.4±0.6Aa	20.8±0.2Bcd	19.1±1.6Ad
Linalool	781.5±15Aa	790.6±12.9Aa	794.6±25.5Aa	779.7±25.8Aa	763±13.2Aa	755.9±16.7Aa
Sum of terpenes	2985.7±27.7Aa	2953.7±29.6Aa	2934.7±60.2Aa	2959.9±51.4Aa	2916.8±26.7Aa	2870.9±29.8Aa
Carboxylic acids						
Acetic acid	59.2±3.1Ab	66.5±1.6Aa	67.6±1Aa	52.9±0.5Ac	50.8±0.2Bc	41.6±2.4Bd
Butanoic acid	433.8±19Aa	429.4±13.2Aa	430±17.6Aa	436.2±12.5Aa	438.5±16.3Aa	442.9±13.7Aa
Hexanoic acid	235.1±2.8Aab	229.9±6.5Ab	234.3±4.4Aab	234.4±4.9Aab	237±12.5Aab	255.1±12.7Aa
Octanoic acid	26.1±2.0Aa	21.2±1.8Bb	23.7±0.7Aab	22.6±0.7abc	20.7±1.0Bc	19.4±1.1Ac
Sum of carboxylic acids	754.2±19.6Aa	746.9±14.9Aa	755.5±18.2Aa	746.1±13.4Aa	747±20.6Aa	759±18.9Aa
Others						
2-Heptanone	22.9±1Aa	20.1±1Bab	22±1.8Ba	20.4±1Aab	22.8±1Aa	17.6±1.9Bb
Ethylbenzene	414.8±9.5Bb	477.1±16.5Aa	480.6±21Aa	484.7±25.9Aa	476.4±13Aa	475.8±20.2Aa
Methional	6.4±0.6Aa	5.8±1.3Aa	5.2±0.4Aa	5.7±0.3Aa	5.9±0.5Aa	6.1±0.0Ba
Eugenol	176.5±12.8Aab	176.1±11.6Aab	173.4±9.9Aab	182.1±6.3Aa	159.5±5.3ABab	153±2.6Ab
Sum of volatile compounds	5246.3±43.9Aa	5268±39.6Aa	5205.5±71Aa	5256.6±62.8Aa	5202.3±38.5Aa	5161.7±68.4Aa
			PEP2			
Aldehydes						
Pentanal	17.1±1.1Cb	30.4±3.1Ba	34±0.4Aa	33.1±1.4Aa	30.8±2.3Aa	35±0.8Aa
Hexanal	6.1±0.3Ab	10.1±1.3Aa	10.4±0.1Ba	10.8±0.1Ba	11.2±0.2Ba	12.8±0.7Ca
(E)-2-Hexenal	397.9±11.2Aa	385.2±7.2Bab	381.9±7.2Aab	375.8±10.7Aab	372.5±2.2Ab	374.7±11.4Aab
(E)-2-Heptenal	8.1±0.5Bd	11.1±0.5Ac	21.3±0.4Ab	12.8±1.8ABc	25±1ABa	25.9±1.2Ca
Nonanal	1.7±0.1Aa	1.7±0.1Aa	1.7±0.3Aa	1.8±0.1Aa	1.7±0.2Ba	2.0±0.0Ba
Benzaldehyde	8.7±0.3Aa	8.3±0.4Aa	8.8±0.5Aa	8.7±0.1Aa	8.7±0.4Aa	8.8±0.2Aa
Sum of aldehydes	439.6±11.3Aa	446.7±8Aa	458±7.2Aa	442.9±11Aa	449.9±3.4Aa	459±11.6Ba
Alcohols						
3-Methyl-1-butanol	18.4±1.2Aa	17.4±0.5Aa	17.5±0Ba	17.7±0.1Aa	17±1.9Aa	17.3±1.3Aa
1-Pentanol	39.2±0.8Ba	36.6±0.6Bab	34.8±0.4Abc	34.7±0.8ABbc	35.4±0.3Bbc	34.4±1.2Ac

1-Octen-3-ol	180.3±8.6Aa	174.1±8.6Aa	174.9±3.9Aa	175.2±11.2Aa	167.1±3.6Ba	167.3±4.3Aa
1-Hexanol	22.5±1Aab	24.5±0.6Aa	21.2±0.1Aab	20.3±1.4Bb	21.5±0.3Aab	20.7±2.5Ab
Benzyl alcohol	10.6±0.5Aa	10±0.4Aa	10.7±0.2Aa	10±0.1Aa	10.3±0.4Aa	9.9±0.6Aa
Phenylethyl alcohol	8.3±0.4Aa	8±0.5Aab	8.3±0.2Aa	8.2±0Aab	8.2±0.2Aab	7.3±0.4Ab
Sum of alcohols	279.2±8.8Aa	270.5±8.6Aa	267.4±3.9Aa	266.1±11.3Aa	259.3±4.1Aa	256.8±5.4Aa
Esters						
Methyl butanoate	21.8±1.2Aa	21.1±0.2Ba	22.9±1.3Aa	20.7±2Aa	20.5±0.7Aa	21.3±1ABa
Ethyl butanoate	58.6±3.0Ab	60.9±1.0Aab	64.3±0.1Aa	63.7±0.1ABa	57.5±0.3Ac	54.5±3.5Ac
Ethyl hexanoate	53.6±4.1Aab	50.1±4.8Aab	53.3±2.1Aab	59.3±4.1Aa	47.4±1.6Ab	46.5±2.2Bb
Ethyl octanoate	11.3±0.9Aa	8.7±2.1Aab	8.0±0.9Ab	8.0±0.5Bb	8.2±0.1Ab	8.6±0.1Aab
Sum of esters	145.3±5.3Ba	140.7±5.4Ab	148.5±2.6Bb	151.7±4.6Ab	133.6±1.8Ab	130.8±4.3Ab
Terpenes						
α-Pinene	230.1±9.6Ca	229.9±1Ca	230.2±12.6Aa	239.5±0.3Aa	228.2±0.1Ca	225.3±2.9Aa
Camphene	16.6±0.3Cb	17.3±0.3Bb	20.8±0.5Aa	20±0.2Ba	18.1±0Bb	17.9±0.9Ab
β-Pinene	178.5±4.3Ba	185.4±0.5Aa	196.7±9.8Aa	186.2±1.7Aa	185.6±0.3Ba	187.5±10.4Aa
β-Thujene	113±1.5Aa	127.4±3.3Aa	105.8±1.5Ca	104.9±0.6Ba	103.9±8.2Ba	100.3±4.9Ba
Sabinene	117.8±2.1Ba	113.4±1.5Cab	103.4±3.9Cb	106.2±1.9Cb	117±0.1Aa	110.6±7.9Aab
β-Myrcene	468.6±34.2Aa	473.7±22.9Aa	428.4±7.5Aa	427.0±33.2Aa	428.4±2Aa	435.1±22.7Aa
Limonene	114.5±7.2Bb	112.5±4.1Cb	136.5±1.2Aa	126.9±7.2Aab	136.8±0.4Aa	134.1±6.8Aa
Eucalyptol	637.4±18.3Aa	620.7±13.9Ba	658±7.1Aa	646.1±9.0Aa	650.9±0.5Aa	649±26.5Aa
β-Ocimene	289.6±16Aa	297.8±14.8Aa	234.8±8.7Ab	244.5±12.8Ab	233.1±4.8Bb	240.5±13.9Bb
Terpinolene	25.1±1.2Ab	19.8±2.3Bc	29.5±1.5Aa	31.7±1.4Aa	24.2±1.3Ab	18.4±0.4Ac
Linalool	789.2±19.5Aa	772.6±61.1Aa	776±35.8Aa	771.5±7.8Aa	779.2±0.9Aa	774.2±6.8Aa
Sum of terpenes	2980.5±48Aa	2970.5±68.6Aa	2920.1±41.7Aa	2904.3±38.3Aa	2905.3±9.9Aa	2892.8±41.3Aa
Carboxylic acids						
Acetic acid	60.1±1.7Aa	58.4±4.5Bab	59.6±1.8Bab	60.1±4.4Aa	55.3±0.3Abc	49.1±2.4Ac
Butanoic acid	425.3±16.5Aa	428±17.7Aa	422.5±2.7Aa	424.1±11.8Aa	434.1±22.4Aa	447.9±28Aa
Hexanoic acid	233.2±13.8Aa	237.7±18.5Aa	236.2±7.6Aa	233.4±0.6Aa	234.5±8Aa	241±11.3Aa
Octanoic acid	23.2±1.2ABb	29.9±1.2Aa	25.1±2.4Aab	23.5±2.8b	26.2±2.4Aab	20.8±0.9Ac
Sum of carboxylic acids	741.8±21.6Aa	754±26Aa	743.4±8.6Aa	741.1±12.9Aa	750±23.9Aa	758.8±30.3Aa
Others						
2-Heptanone	21.4±1.3ABab	24.4±1.5Aa	24±0.4Aab	20.2±0.7Ab	21.6±1.0Aab	20.8±2.8Aab
Ethylbenzene	417.1±14.3Bb	407.1±11.5Bc	412.1±13.1Bb	422.1±12.6Babc	457.3±14.3Aab	463.5±27.5Aa
Methional	6.3±0.6Aa	6.0±0.4Aa	6.3±0.2Aa	6.8±0.4Aa	6.6±0.6Aa	7.2±0.2Aa
Eugenol	180.6±0.6Aa	177.3±11.5Aa	175.1±14.6Aa	175.7±8.5ABa	180±15.4Aa	163.3±9.1Aa
Sum of volatile compounds	5211.8±56.7Aa	5197.4±76.2Aa	5154.8±47.7Aa	5130.8±46.2Aa	5163.6±33.9Aa	5153.0±60.4Aa

* Results are the mean of two independent analytical determinations ± standard deviation. Different capital letters (A–C) in the column within each storage day represent significant differences among

the formulations ($p < 0.05$); different lowercase letters (a–d) in the row within each formulation represent significant differences as a function of storage days after opening ($p < 0.05$). Legend: CTRL, control plus 0.06 g ascorbic acid/kg pesto and 1 g sorbic acid/kg pesto; PEP1, plus PE corresponding to 250 mg phenols/kg pesto; and PEP2, plus PE corresponding to 500 mg phenols/kg pesto.

Table S6. Colour variation (ΔE) in pesto samples during the SSL (at opening (day 0) and 3 and 7 days of storage after opening).

Days of storage	CTRL vs. PEP1	CTRL vs. PEP2	PEP1 vs. PEP2
0	2.0±0.1	1.6±0.1	0.6±0.0
3	3.8±0.0	0.3±0.0	3.8±0.1
7	2.3±0.0	6.0±0.0	4.0±0.0

The results are the mean of two independent analytical determinations ± standard deviation. Legend: CTRL, control plus 0.06 g of ascorbic acid/kg of pesto and 1 g of sorbic acid/kg of pesto; PEP1, plus PE corresponding to 250 mg of phenols/kg of pesto; and PEP2, plus PE corresponding to 500 mg of phenols/kg of pesto.
